# Histone modifications associated with gene expression and genome accessibility are dynamically enriched at *Plasmodium falciparum* regulatory sequences

**DOI:** 10.1186/s13072-020-00365-5

**Published:** 2020-11-23

**Authors:** Jingyi Tang, Scott A. Chisholm, Lee M. Yeoh, Paul R. Gilson, Anthony T. Papenfuss, Karen P. Day, Michaela Petter, Michael F. Duffy

**Affiliations:** 1 Department of Medicine, The University of Melbourne, Royal Melbourne Hospital, Parkville, VIC 3050 Australia; 2 School of Medicine, Faculty of Health, Deakin University, Geelong Waurn Ponds Campus, Waurn Ponds, VIC 3216 Australia; 3 School of BioSciences, The University of Melbourne, Parkville, VIC 3052 Australia; 4 Bio21 Institute, Parkville, VIC 3052 Australia; 5 Peter Doherty Institute, Melbourne, VIC 3000 Australia; 6 Department of Microbiology and Immunology, The University of Melbourne, Victoria, 3000 Australia; 7 Macfarlane Burnet Institute for Medical Research and Public Health, Melbourne, VIC 3004 Australia; 8 Monash University, Melbourne, VIC 3800 Australia; 9 Bioinformatics Division, Walter and Eliza Hall Institute of Medical Research, Parkville, VIC Australia; 10 Department of Mathematics and Statistics, University of Melbourne, Victoria, Australia; 11 Department of Medical Biology, University of Melbourne, Parkville, VIC Australia; 12 Sir Peter MacCallum, Department of Oncology, University of Melbourne, Parkville, VIC Australia; 13 Erlangen University, 91054 Erlangen, Germany

**Keywords:** *Plasmodium falciparum*, Histone modifications, Gene regulation

## Abstract

**Background:**

The malaria parasite *Plasmodium falciparum* has an unusually euchromatic genome with poorly conserved positioning of nucleosomes in intergenic sequences and poorly understood mechanisms of gene regulation. Variant histones and histone modifications determine nucleosome stability and recruit *trans* factors, but their combinatorial contribution to gene regulation is unclear.

**Results:**

Here, we show that the histone H3 acetylations H3K18ac and H3K27ac and the variant histone Pf H2A.Z are enriched together at regulatory sites upstream of genes. H3K18ac and H3K27ac together dynamically mark regulatory regions of genes expressed during the asexual life cycle. In contrast, H3K4me1 is depleted in intergenic sequence and dynamically depleted upstream of expressed genes. The temporal pattern of H3K27ac and H3K18ac enrichment indicates that they accumulate during S phase and mitosis and are retained at regulatory sequences until at least G1 phase and after cessation of expression of the cognate genes. We integrated our ChIPseq data with existing datasets to show that in schizont stages H3K18ac, H3K27ac and Pf H2A.Z colocalise with the transcription factor PfAP2-I and the bromodomain protein PfBDP1 and are enriched at stably positioned nucleosomes within regions of exposed DNA at active transcriptional start sites. Using transient transfections we showed that sequences enriched with colocalised H3K18ac, H3K27ac and Pf H2A.Z possess promoter activity in schizont stages, but no enhancer-like activity.

**Conclusions:**

The dynamic H3 acetylations define *P. falciparum* regulatory sequences and contribute to gene activation. These findings expand the knowledge of the chromatin landscape that regulates gene expression in *P. falciparum*.

## Background

The parasite *Plasmodium falciparum* is the cause of most global malaria mortality. *P. falciparum* differs from other eukaryotes in the unusually high AT content of its genome (90% in intergenic regions) [1], its unique variant histones [2] and the high proportion of its genome that it maintains as euchromatin during its pathogenic, intra-erythrocytic lifecycle [3]. Structural analysis of *P. falciparum* nucleosomes confirmed the unique nature of *P. falciparum* chromatin [4], which may therefore exert novel mechanisms of control over gene expression. The majority of *P. falciparum* genes are specifically regulated during its haploid, 48-h intra-erythrocytic developmental cycle (IDC) [5, 6] and groups of *Plasmodium* genes are specifically activated by stage-specific transcription factors [7]. In at least one instance a stage-specific transcription factor [8] requires the presence of the *P. falciparum* bromodomain protein 1 (PfBDP1) to activate a subset of erythrocyte invasion genes [9]. PfBDP1 binds acetylated lysines in histones and presumably acts as a transcriptional co-factor. Thus, the many possible combinations between chromatin-binding proteins and specific transcription factors may explain the tightly regulated, dynamic transcription of most *P. falciparum* genes.

Increased nucleosome occupancy can directly hinder transcription and is dependent on nucleosome stability and thus nucleosome composition. Histone modifications can also recruit factors that facilitate or repress transcription. Associations between combinations of variant histones and histone modifications with depletion of nucleosomes at regulatory elements have been shown in yeast and for the human Encyclopedia of DNA Elements (ENCODE) Project. Yeast and human active promoters are enriched in H3K9ac, H3K18ac, H3K27ac, H3K4me3 and the histone variant H2A.Z [10–20], whereas active human enhancer elements are characterised by high levels of H3K4me1, H3K18ac, H3K27ac and H2A.Z [10, 11, 16, 20, 21]. H2A.Z enrichment levels correlate with human enhancer strength [18, 22] and H3K27ac enrichment levels differentiate active from inactive metazoan enhancers [23, 24]. The transcriptional co-activators and histone acetyltransferases p300/CBP responsible for acetylating H3K27 and H3K18 [25] are also enriched at mammalian enhancers [11, 26–28]. Nucleosomal occupancy is relatively depleted around human promoters, but both nucleosomal occupancy and positioning is maintained around the exposed transcription factor binding site at putative human enhancers [29].

Metazoan enhancers can be many kb distant from the cognate gene, but yeast have upstream activating sequences (UAS) that are typically located within 450 bp of the transcriptional start site (TSS) [30]. Analogous to distal metazoan enhancers, UASs bind the essential, transcriptional regulator mediator complex [31], specific transcription factors, and chromatin-modifying complexes [32, 33] to regulate expression of the core promoter. Yeast maintain 2–3 nucleosomes in the 500 bp upstream of the TSS [34, 35]. However, gene activation is associated with depletion of the consistently positioned -1 nucleosome at approximately -250 bp [35] and in ribosomal protein genes a nucleosome depleted region extends from the start codon to the UAS [33].

Throughout the *P. falciparum* intra-erythrocytic cycle gene expression correlates with intergenic, upstream, dynamic enrichment of several euchromatic histone modifications [3, 36–39]. These include enrichment of H3K9ac, H3K4me3 and Pf H2A.Z flanking the regions of greater chromatin accessibility that define promoter sequences and specific transcription factor binding sites [40, 41].

Multiple regions of increased chromatin accessibility [40, 41] and multiple transcriptional start sites (TSSs) [42, 43] are found within the same *P. falciparum* intergenic regions. *P. falciparum* intergenic sequence is maintained in a nucleosomal array aside from a short, nucleosome depleted region (NDR) of variable length that precedes the well positioned + 1 nucleosome at TSSs [43]. Whether the multiple TSSs function in the same cell and whether they mark regulatory elements that have a synergistic or antagonistic effect on the core promoter or are simply promiscuous TSSs is unknown. The average intergenic size of approximately 1500 bp in *P. falciparum* is too short to resolve enhancer and promoter interactions within the same intergenic region using Hi-C approaches [44] and more distant interactions have not been detected outside of the contacts due to clustering of rRNA genes and virulence genes, suggesting distant enhancers do not exist in *P. falciparum* [45–47]. Putative *P. falciparum* enhancer-like elements identified by relative H3K4me1 enrichment [48] did not correlate with putative transcription factor binding sites identified by ATACseq (Assay for Transposase Accessible Chromatin using sequencing) [40]. H3K18ac and H3K27ac are enriched at both metazoan enhancers and promoters so they could be enriched at all regulatory sequences in *P. falciparum.* Indeed H3K27ac is enriched in intergenic sequence of euchromatic genes in *P. falciparum* oocysts and sporozoites, but is not clearly associated with gene expression in these mosquito stages [49].

To get better insight into the complex regulatory network underlying *P. falciparum* gene expression we investigated the genome-wide correlation of promoter and enhancer associated histone modifications with cognate gene expression profiles as well as with published DNA accessibility and transcription factor binding data. We demonstrate that dynamic enrichment of H3K18ac and H3K27ac at Pf H2A.Z containing nucleosomes at TSSs correlates with gene expression levels, whereas H3K4me1 is generally absent from intergenic sequences and thus depleted from these putative regulatory elements. Acetylations of H3K18 and H3K27 accumulate during mitosis and appear to mark regulatory sequences of genes for expression rather than as a consequence of expression. From transient transfection analysis, we concluded that histone modifications that identify active enhancers and promoters in metazoan and yeast cells do identify *P. falciparum* sequences with schizont stage promoter activity but not enhancer-like elements.

## Results

To investigate chromatin structure of the *P. falciparum* genome and how this is associated with regulatory sequences and gene expression, we analysed genomic enrichment of the variant histone H2A.Z and the histone modifications H3K4me1, H3K18ac and H3K27ac. These markers of chromatin structure are all enriched at metazoan enhancers and, with the exception of H3K4me1, at yeast and metazoan promoters [10–24].

### Levels of H3K4me1 and Pf H2A.Z remain constant across the intra-erythrocytic development cycle (IDC) but levels of H3K18ac and H3K27ac vary

The antibodies we used to investigate the chromatin marks included a Pf H2A.Z antiserum of confirmed specificity [50] and commercial antibodies to H3K4me1, H3K27ac and H3K18ac that were previously validated for chromatin immunoprecipitation (ChIP) (Antibody Validation Database (https://compbio.med.harvard.edu/antibodies/)) [51], (Histone Antibody Specificity Database (https://www.histoneantibodies.com)) [52]. We confirmed specificity of these anti-human histone antibodies for *P. falciparum* histones by showing that each antibody bound only the single H3 band in western blots of *P. falciparum* histones (Additional file 1: Fig S1). We also showed their differential binding to dot blots of ten custom-made biotinylated *P. falciparum* peptides and six commercially available human peptides that were largely conserved with *P. falciparum* (Additional file 2: Fig S2). The anti-H3K18ac, anti-H3K27ac and anti-H3K4me1 antibodies all bound to their cognate histone modification with greater than 32-fold sensitivity compared to background, well over the ENCODE standard that signals from the cognate peptide should be at least tenfold higher than those from other peptides [53].

**Fig. 1:**
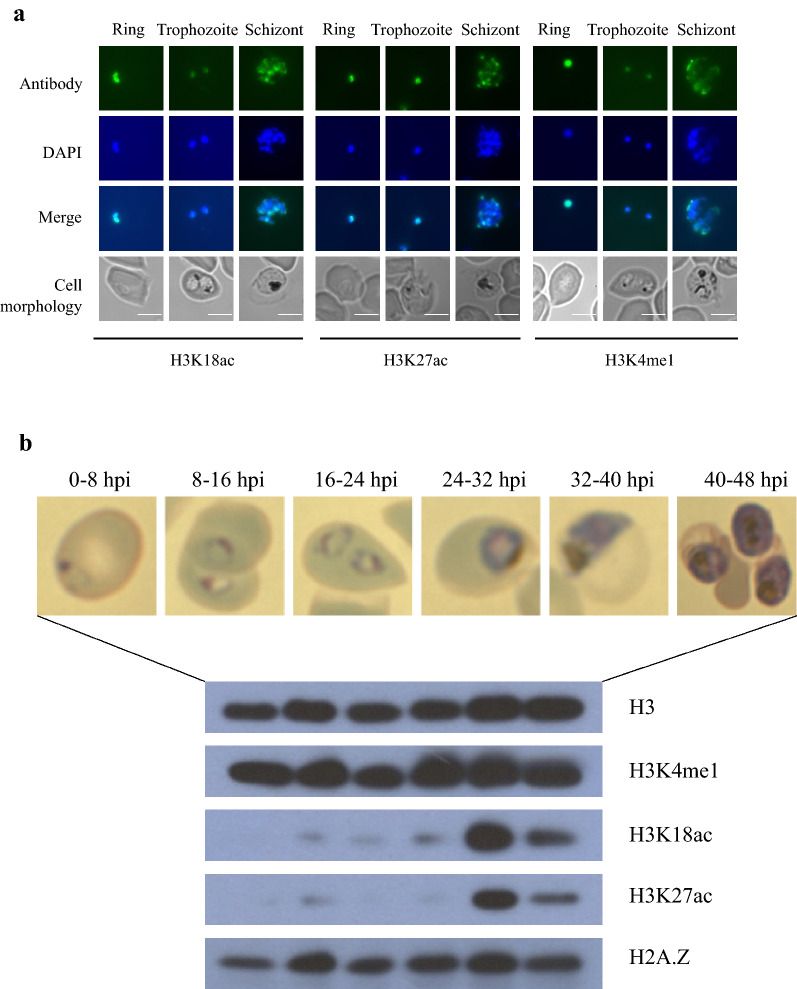
Distribution of histones and their modifications throughout the intra-erythrocytic developmental cycle. **a** Immunofluorescence assay shows that PfH3K18ac, PfH3K27ac and PfH3K4me1 localise to the nucleus of *P. falciparum* in ring-, trophozoite- and schizont-stages in the IDC. Bound antibody shown in green in the first row was specific for the histone modifications indicated at the bottom of the figure. DNA was stained blue with DAPI in the second row. Bright field microscopy shows the cell morphology in the fourth row. Scale bar is 5 μm. **b** *P. falciparum* H3K4me1, H3K18ac, H3K27ac and H2A.Z levels at 0–8 hpi, 8–16 hpi, 16–24 hpi, 24–32 hpi, 32–40 hpi and 40–48 hpi during the IDC. Cell morphology at the six time points when the parasite lysates were prepared is indicated on top. H3 levels were shown as the parasite lysate loading control

Fluorescence microscopy confirmed the predicted nuclear localisation of all three H3 modifications (Fig. 1a). The diffuse signal from anti-H3K18ac, anti-H3K27ac and anti-H3K4me1 largely colocalised with the DAPI-stained nucleus in ring and trophozoite stages.

The abundance of Pf H2A.Z and the H3 modifications throughout the intra-erythrocytic lifecycle was assessed by western blot using histone H3 as a loading control. The levels of H3K4me1 and H2A.Z were constant throughout the lifecycle. The levels of both H3K18ac and H3K27ac were low in ring- to mid-trophozoite-stages and peaked at late-trophozoite- to early-schizont-stages (32–40 hpi) prior to dropping at mid- to late-schizont stage (Fig. 1b). This was consistent with previous western blot and mass spectrometry data [54, 55] and correlated with the total transcriptional activity that peaks at late stage in the IDC [56], and indicated that H3K18ac and H3K27ac may contribute to regulation of transcription.

### Pf H2A.Z, H3, H3K18ac and H3K27ac are differentially enriched across the genome

ChIPseq of Pf H2A.Z, H3K18ac, H3K27ac, H3K4me1 and H3 was performed in ring, trophozoite and schizont-stage parasites in biological duplicates as per ENCODE guidelines [53, 57, 58], and matched input was also sequenced. Matched RNAseq was performed for each duplicate chromatin sample and trophozoite and schizont-stage analyses were augmented with an additional RNA biological replicate. For both RNAseq and ChIPseq the average ± standard deviation quality (all more than 86.4 ± 3.6% reads ≥ Q30) and coverage (all more than 94.3 ± 0.7% of reads mapped to *P. falciparum*, all greater than 27 ± 8 X genome coverage) of the libraries were all acceptable (Additional files 3, 4: Tables S1 and S2) and the biological replicates clustered together by principal component analysis (PCA) (Additional file 5: Fig S3) and were clearly reproducible (Additional file 6: Fig S4). The input clustered with H3K4me1 and H3, whereas H3K18ac, H3K27ac and Pf H2A.Z formed a separate cluster. The GC sequencing bias was controlled for by normalising to input.

**Fig. 2:**
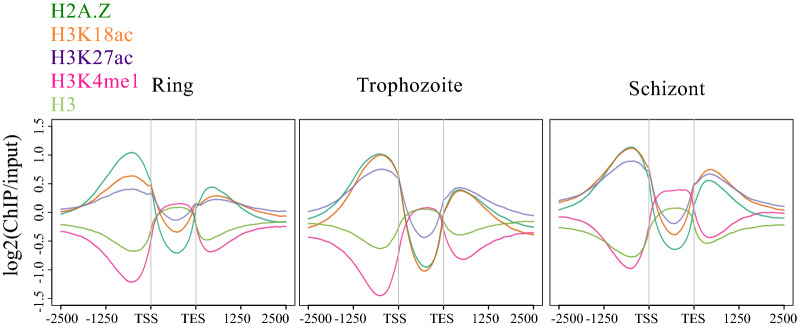
Enrichment profiles of histones and their modifications relative to transcriptional units. Average ± SE enrichment profiles (log2 ratio of ChIP over stage-matched input) of H2A.Z (dark green), H3K18ac (orange), H3K27ac (purple), H3K4me1 (pink) and H3 (pale green) plotted across all transcripts from the transcription start sites (TSSs) to the transcription end sites (TESs) with an extension of 2500 bp upstream and downstream in ring, trophozoite stage and schizont stages

In all three lifecycle stages Pf H2A.Z, H3K18ac and H3K27ac were globally enriched upstream of transcriptional start sites and downstream of transcriptional end sites (TESs), but depleted within transcripts determined from the cognate RNAseq (Fig. 2). The intergenic enrichment of H3K18ac and H3K27ac was greater in trophozoites (30–36 hpi) and schizonts (38–44 hpi) than in ring stages, consistent with the western blot data for whole-cell levels of these modifications that peaked at 32–40 hpi. Conversely, H3 and H3K4me1 were depleted up and downstream of transcripts, but were relatively enriched in the transcribed sequences. This explains H3 and H3K4me1 clustering in PCA with input, which has a slight sequencing bias towards coding sequence.

### Pf H2A.Z, H3K18ac, H3K27ac, H3K4me1 and unmodified H3 enrichment vary with gene expression

**Fig. 3:**
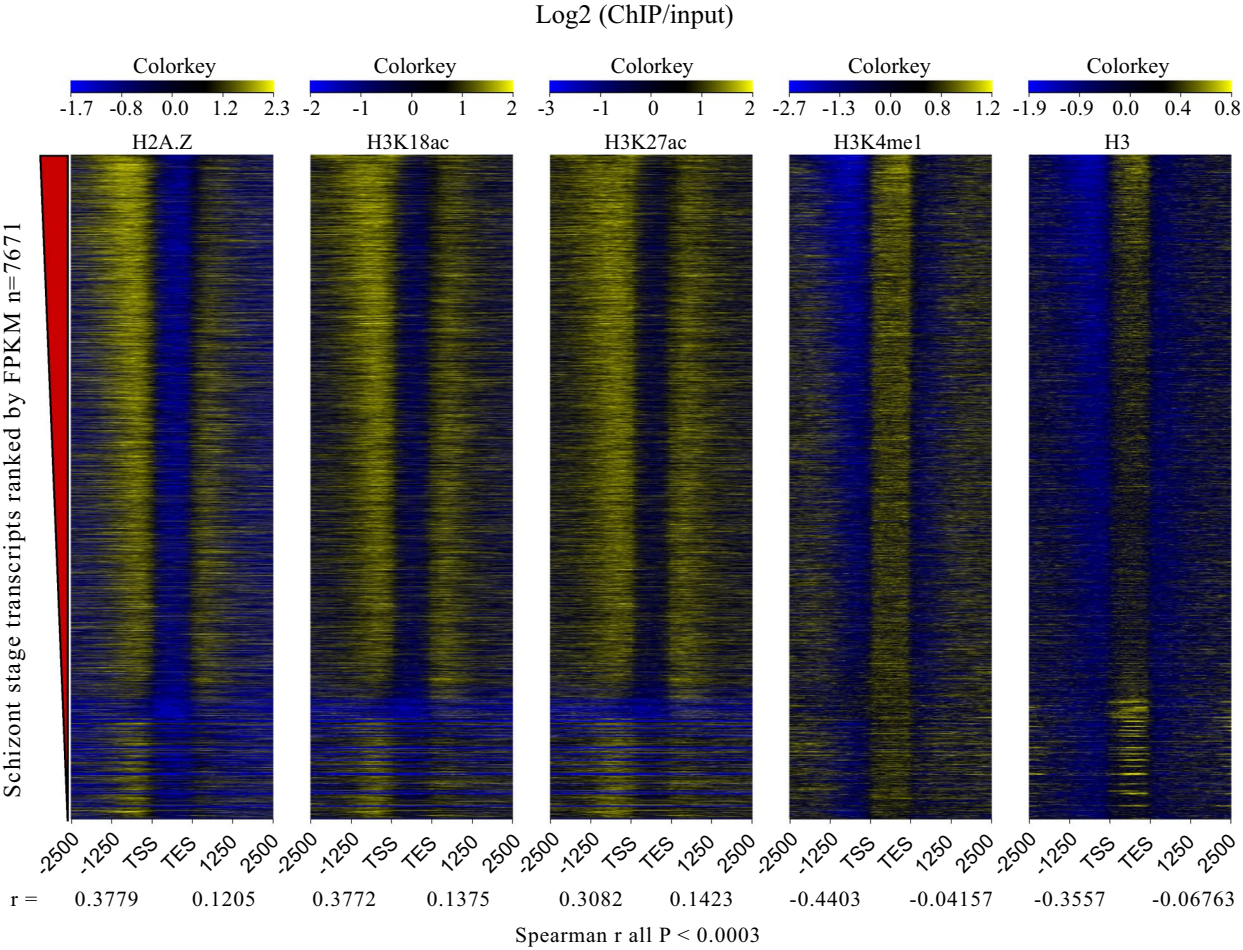
Correlations between histones and their modifications and transcription. Log2 (ChIP/input) plotted from 2500 bp upstream (-2500) to 2500 bp downstream (2500) of the transcriptional start (TSS) and stop (TES) sites for all 7671 schizont-stage transcripts assembled by Cufflinks ranked in descending order by transcript abundance (fpkm). The Spearman correlation value (r) for the upstream and downstream enrichment of each histone or histone modification is indicated below the plots

Ranking genes by expression levels demonstrated that the upstream enrichment of Pf H2A.Z, H3K18ac and H3K27ac positively correlated with gene expression in all three lifecycle stages. Conversely, the abundance of H3 and H3K4me1 upstream of coding sequences inversely correlated with transcript levels in all three lifecycle stages (Fig. 3, Additional file 7: Fig S5) (all Spearman r correlations and p values are provided in the figures).

The depletion of intergenic H3 was consistent with the correlation between intergenic nucleosome depletion and gene expression in *P. falciparum* [43]. Silent genes and genes in the lowest tercile by expression had very similar upstream, intergenic profiles of moderate depletion of H3K4me1 and unmodified H3, suggesting that H3K4me1 levels simply mirrored nucleosomal occupancy for these genes. However, genes in the top tercile by expression were markedly depleted of upstream H3K4me1 compared to H3 (Additional file 8: Fig S6 D,E,I,J,N,O), indicating active depletion of H3K4me1 at the promoter of highly expressed genes. This would be consistent with additional methylation of H3K4 and previous reports of H3K4me3 enrichment at regulatory sequences [3, 36, 38].

**Fig. 4:**
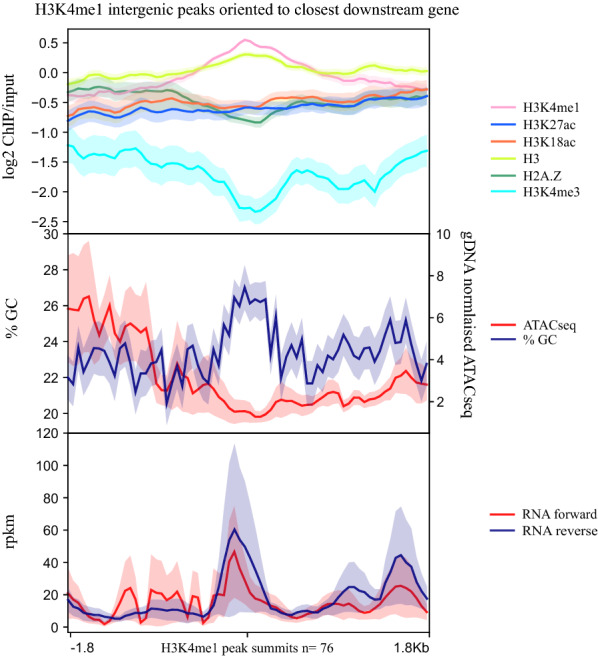
Average enrichment profiles of chromatin features and RNA levels across intergenic, H3K4me1 peaks. Average profile plots ± SE covering 1.8 kb up and downstream of the summit of H3K4me1 intergenic peaks (*n* = 76) oriented according to the closest downstream gene. Top panel is the log2 ratio of ChIP over input average enrichment profiles of H3K4me1, H3K27ac, H3K18ac. H3 and Pf H2A.Z, all from this study and also log2 ratio of H3K4me3 ChIP over input from [38]. Middle panel is GC content and ATACseq normalised to gDNA and the bottom panel is RNA separated by strand

Although on average intergenic H3K4me1 was depleted, 76 intergenic peaks of H3K4me1 were detected in schizont stages. Bidirectional transcription originated from the H3K4me1 peaks and the peaks also colocalised with relative depletion of H3K4me3 in a published schizont dataset [38] (Fig. 4), which are characteristics of some enhancer sequences [11]. However, H3 levels were increased at these peaks while DNA accessibility was low, as indicated by published ATACseq signals [41] (Fig. 4); both of these properties were consistent with increased nucleosomal occupancy and thus inconsistent with enhancer function. Furthermore, Pf H2A.Z, H3K27ac and H3K18ac were not enriched at the intergenic H3K4me1 peaks and transcription of the closest, downstream genes (median 11.7 rpkm IQR 1.4, 70.5 rpkm) was similar to the average of all the other protein coding genes in the *P. falciparum* genome in schizonts (median 19.9 IQR 3, 72.8 rpkm Mann–Whitney test *p* = 0.2636). Interestingly, GC content was very high for intergenic sequence.

To further investigate whether the H3K4me1/H3K4me3 ratio was informative of putative enhancer function, we identified peaks of H3K4me1 calculated relative to published H3K4me3 (Additional file 9: Fig S7A) [38]. This set of peaks included most of the H3K4me1 peaks identified by comparison to input (Additional file 9: Fig S7C) and had a similar profile (Additional file 9: Fig S7A) for all characteristics described above except that the adjacent downstream genes were expressed at significantly lower levels (median 8.9 IQR 0.7, 43.1 rpkm Mann–Whitney *p* = 0.0009) than all of the other protein coding genes in the *P. falciparum* genome in schizonts. Overall, the H3K4me1/input and H3K4me1/H3K4me3 data suggested that intergenic H3K4me1 enrichment was not marking transcription regulating sequences. Indeed, the AT content and chromatin characteristics of these peaks were more similar to coding than intergenic sequence.

A recent study reported that intergenic enrichment of H3K4me1 in *P. falciparum* marked regulatory regions [48]. In this report regions of H3K4me1 enrichment were divided into “strong” and “weak” peaks, but only 259 of the 462 “strong” peaks were longer than one nucleotide. None of the H3K4me1 intergenic peaks we detected overlapped with these 259 previously reported peaks (Additional file 9: Fig S7C) [48]. However, H3K4me1 was also enriched relative to adjacent intergenic sequence in our ChIP data at these 259 sites, albeit at a lower level than at the intergenic H3K4me1 peaks we found de novo, probably reflecting natural variation between the parasites (Additional file 9: Fig S7B). Although not highly correlated (Additional file 9: Fig S7D), the broad chromatin and transcriptional patterns of the published 259 H3K4me1 intergenic peaks (Additional file 9: Fig S7B) were similar to those of the intergenic H3K4me1 peaks discovered de novo in this study (Fig. 4), suggesting similar features were detected in both studies.

### H3K18ac and H3K27ac upstream enrichment dynamically correlates with expression of genes upregulated in mature parasites

*P. falciparum* genes are dynamically regulated across the IDC. Transcription of highly expressed genes can still vary through the cycle, but even at their minimum level of expression many may still be in a high percentile of genes by transcript level. Thus the correlation between steady-state mRNA levels and upstream enrichment of a histone mark may not reflect a dynamic association with gene expression, as has been noted before for Pf H2A.Z [59]. To assess whether Pf H2A.Z, H3K27ac, H3K18ac and H3K4me1 were dynamically associated with gene expression, their enrichment was examined across the coding sequences and for 2500 bp upstream and downstream of genes that were differentially expressed throughout the IDC. Average enrichment profiles were plotted for gene sets that were expressed in the top quartile in a particular IDC stage and were expressed at least threefold more in that stage than in another, compared IDC stage (Additional file 10: Table S3). The stage-specific transcription of these genes was confirmed using a published nascent RNA dataset [60] (Additional file 11).

**Fig. 5:**
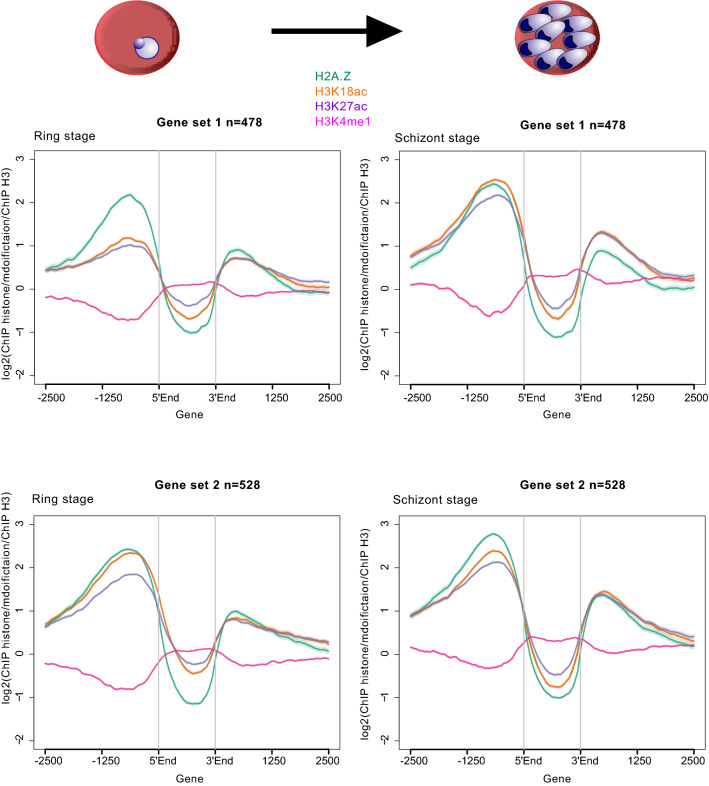
Comparison of enrichment profiles of histones and their modifications across genes dynamically expressed in rings and schizonts. Average log2 ratio of ChIP enrichment of Pf H2A.Z (green), H3K18ac (orange), H3K27ac (purple) and H3K4me1 (pink) all relative to ChIP enrichment of H3 plotted over the coding sequences ± 2500 bp of: gene set 1 (*n* = 478) that were expressed at least threefold more in schizont stage than in ring stage and that were in the top quartile by schizont-stage expression and: gene set 2 (*n* = 528) that were expressed at least threefold more in ring stage than in schizont stage and that were in the top quartile by ring-stage expression. 5′End: the start codon of a gene; 3′End: the stop codon of a gene

H3 is depleted in non-coding sequence due either to decreased nucleosomal occupancy [61] or to technical artefacts relating to the high AT content of intergenic sequence affecting nucleosome stability during experimental procedures [43]. Although our H3 ChIP protocol cannot distinguish between these alternatives, we did control for the level of histone modifications per nucleosome by normalising to H3 levels. Upstream, intergenic enrichment of all three H3 modifications and H2A.Z were statistically different between stages except for H3K18ac levels in trophozoite stage-expressed genes, which were not different between trophozoite and schizont stages (Wilcoxon matched-pairs signed rank test, Additional file 12: Table S4). However, the variation in the magnitude of these differences suggested some were not biologically significant. H3K4me1 was depleted upstream of dynamically regulated genes regardless of their expression level (Fig. 5, Additional file 13, 14: Fig S8, S9). Compared to ring stages, H3K18ac and H3K27ac levels clearly increased in trophozoites and schizonts upstream of genes that had peak expression in mature trophozoite and schizont-stage parasites, whereas Pf H2A.Z levels were steady and did not obviously vary with differential expression (gene set 1, Fig. 5 and gene set 3, Additional file 13: Fig S8). Conversely, Pf H2A.Z, H3K18ac and H3K27ac were all enriched upstream of genes that had peak expression in ring stages with no obvious variation compared to trophozoites and schizonts (gene set 2, Fig. 5, and gene set 4, Additional file 13: Fig S8). The dynamic, expression-associated enrichment was also apparent upstream of schizont-expressed genes when compared to trophozoites, but the level of enrichment was already relatively high in trophozoites (gene set 5, Additional file 14: Fig S9). In contrast, the H3K18ac and H3K27ac enrichment upstream of trophozoite-expressed genes did not obviously decrease in schizonts (gene set 6, Additional file 14: Fig S9).

The patterns of H3K18ac and H3K27ac enrichment may indicate that these histone modifications accumulate during mitosis (approximately 27–40 h post-invasion [62]) in nucleosomes upstream of all genes to be expressed until the next round of replication in the following IDC. They were also consistent with western blot evidence of peak H3K18ac and H3K27ac abundance from 32 to 40 h post-invasion (Fig. 1). The modifications appeared to be removed in G1 phase after invasion, but only upstream of those genes that had been expressed and silenced since the commencement of mitosis, i.e. genes expressed in trophozoites and schizonts. Thus, in ring stages, the histone modifications remained only in ring stage-expressed genes. Therefore, we conclude that these histone modifications precede gene expression and may mark genes for subsequent expression, while their removal (by unknown HDACs) occurs after repression of the downstream gene.

### Colocalised, intergenic peaks of H3K18ac, H3K27ac and Pf H2A.Z overlap, or are close, to putative regulatory regions

The dynamic, enrichment of H3K27ac and H3K18ac upstream of expressed genes in *P. falciparum* was consistent with their collective marking of regulatory sequences, as they do, together with H2AZ, in yeast and metazoans. To investigate this possibility, Pf H2A.Z peaks that intersected H3K18ac and H3K27ac peaks were filtered to retain only the 2196 ring stage and 2645 schizont stage, intergenic, colocalised peaks (ICPs). Existing datasets of nucleosomal occupancy (DNAseq of micrococcal nuclease-treated chromatin) [43], DNA accessibility (ATACseq) [40, 41] and transcriptional start sites [42] were examined for associations with possible regulatory sequences defined by ICPs.

**Fig. 6:**
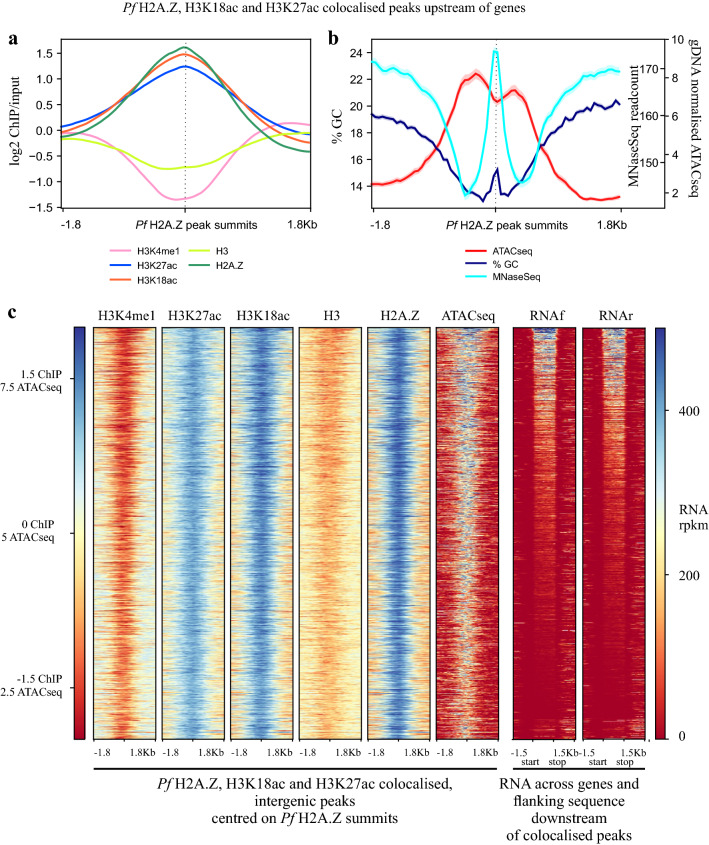
Intergenic, colocalised Pf H2A.Z, H3K18ac and H3K27ac mark well-positioned nucleosomes within a region of exposed DNA. **a** ChIP, **b** % GC, gDNA normalised ATACseq [41], MNaseSeq readcounts [43] and (**c**) ChIPseq, matched, stranded RNASeq rpkm and ATACseq plotted across the 2645 colocalised Pf H2A.Z, H3K18ac and H3K27ac schizont-stage intergenic peaks. The plot profiles and heatmaps are centred on the Pf H2A.Z peak summits. The heatmap was sorted in descending order of the schizont-stage RNAseq rpkm of the closest downstream gene

**Fig. 7:**
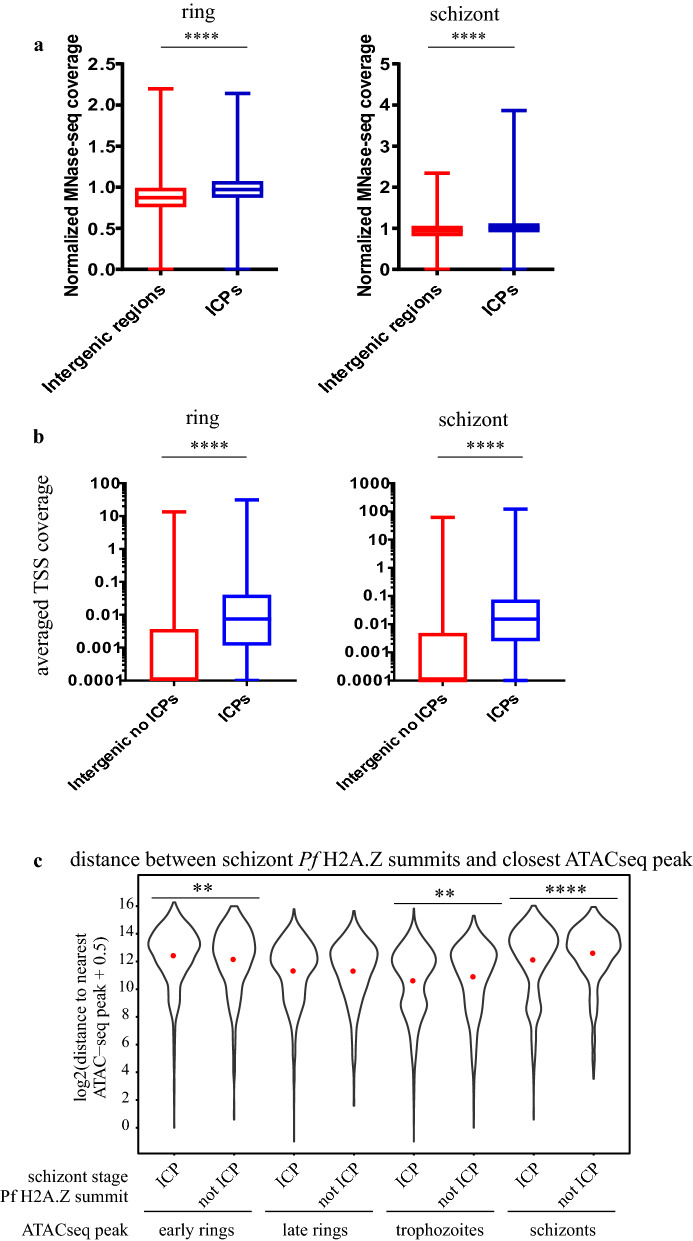
Intergenic, colocalised peaks of Pf H2A.Z, H3K18ac and H3K27ac compared to other intergenic regions have increased nucleosomal occupancy and increased density of TSSs and are closer to regions of exposed DNA. **a** Per site normalised *P. falciparum* MNase-seq read coverage [43] from ring stages (15 h post-invasion) and schizont stages (40 h post-invasion) plotted across all 5567 intergenic regions and across the 2197 ring stage and 2645 schizont stage intergenic, colocalised H2A.Z, H3K18ac and H3K27ac ChIP peaks. Whiskers are minimum and maximum values. Mann Whitney U test ****p < 0.0001. **b** Average, stranded, *P. falciparum* TSS tag coverage at 10 and 42 h post-invasion [42] at ICPs and at all other intergenic regions. (Mann–Whitney U test **** *p* < 0.0001). **c** Distance between ATACseq peaks [40] in early rings, late rings, trophozoites or schizonts and schizont-stage Pf H2A.Z summits of colocalised, intergenic peaks (ICP) or schizont-stage Pf H2A.Z, intergenic summits that did not colocalise with H3K18ac or H3K27ac peaks (not ICP), t test **** *p* < 0.0001, ** *p* < 0.01

The inferred nucleosomes at the Pf H2A.Z summits of the 2645 ICPs in schizont stages (Fig. 6a) overlaid a pronounced peak of nucleosomal occupancy previously mapped by MNaseSeq [43] (Fig. 6b). The nucleosome occupancy at the ICPs was significantly higher than the occupancy at all intergenic regions combined (Fig. 7a). Thus, the nucleosomes defined by the ICPs presumably represented well-positioned nucleosomes because they were conserved between these studies. From matched RNASeq 35% of the 8415 active TSSs intersected with colocalised Pf H2A.Z, H3K18ac and H3K27ac peaks.

The association between the ICPs and TSSs was confirmed with an orthogonal TSS sequence tag dataset [42]. In both ring stages (10 hpi) and schizonts (42 hpi), the ICPs intersected with more TSS sequence tags [42] than did other intergenic sequences (Mann–Whitney U test *p* < 0.0001) (Fig. 7b).

The association between the TSSs and the ICPs suggested an association with regulatory sequences. ATACseq reveals exposed DNA presumed to be available to bind transcription factors. The 2645 schizont-stage ICPs overlaid ATACseq peaks [41] and regions of low GC content (Fig. 6b, c). Similarly, nearly all ATACseq peaks [41] were enriched in H3K27ac, H3K18ac and Pf H2A.Z (Additional file 15: Fig S10), the latter having been shown previously [40]. The Pf H2A.Z summits of the ICPs overlaid a distinct dip in ATACseq reads and a distinct increase in GC content (Fig. 6b), probably reflecting reduced accessibility due to the Pf H2A.Z containing nucleosome positioned at the summit peak, and possibly a role for GC in positioning of the Pf H2A.Z nucleosome. A slight spike in %GC at the *P. falciparum* TSS has been previously noted [43].

In schizont stages the Pf H2A.Z summits of the ICPs were on average 1.3-fold closer (4400 bp) to schizont-stage ATACseq peaks [40] than were summits of intergenic, non-colocalised Pf H2A.Z peaks (5900 bp) (*p* < 0.00005 Welch’s t test with Bonferroni correction) (Fig. 7c). The trophozoite-stage ATACseq peaks were also 1.2-fold closer to schizont ICPs compared to schizont intergenic non-ICPs, but the difference was much less significant (*p* = 0.005) whilst ring-stage ATACseq peaks were not closer to schizont ICPs compared to schizont intergenic non-ICPs. These differences are consistent with a stage-dependent proximity of the ICPs to the stage-matched ATACseq peaks.

**Fig. 8:**
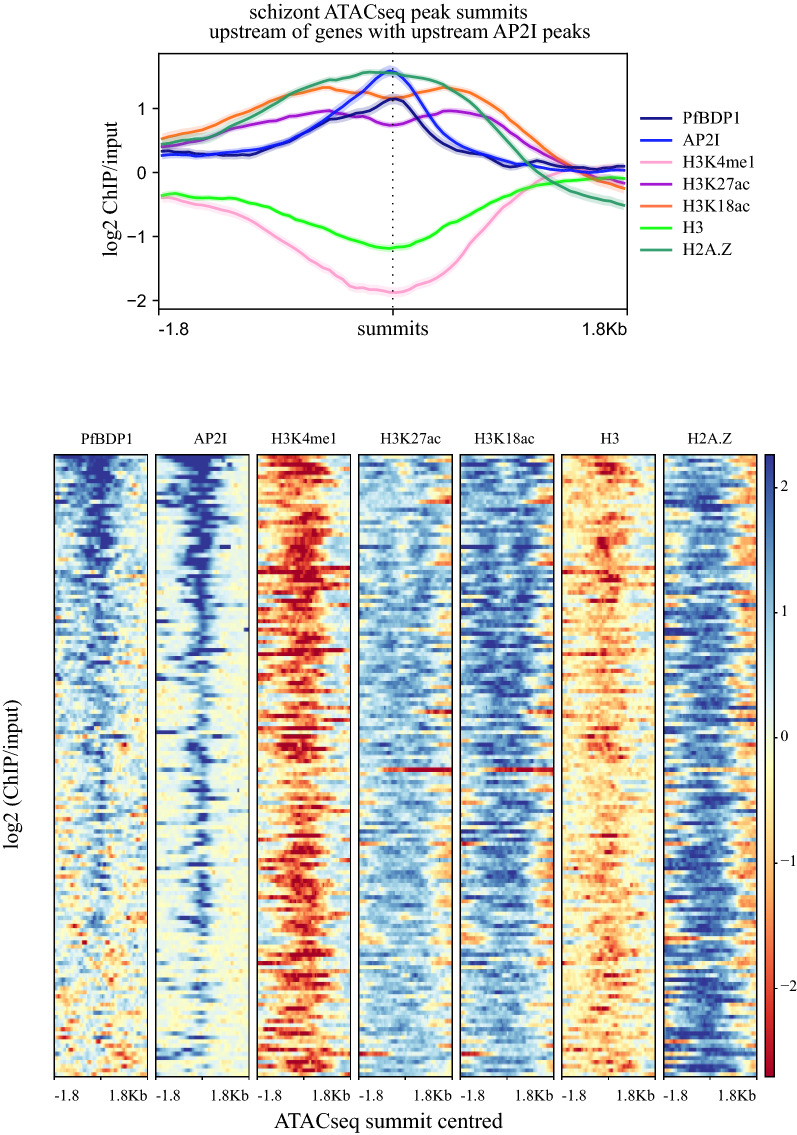
H3K18ac and H3K27ac flank sequences bound by AP2-I and enriched in Pf H2A.Z and PfBDP1. The 151 ATACseq peaks that were closest to, and upstream of, the 151 genes that also had upstream AP2-I and PfBDP1 peaks. ATACseq peak average length was 675 bp. Plots are log2 ChIP/input centred on ATACseq peak summits. Profile plots are average values with standard error of mean shaded. Heatmaps were all sorted by descending level of PfBDP1 enrichment

To further investigate the associations between Pf H2A.Z, H3K27ac, H3K18ac and a transcription factor (PfAP2-I), its binding sites (ATACseq) and co-activator (PfBDP1), the well characterised PfAP2-I/PfBDP1-regulated invasion genes were analysed [8, 9, 41]. Pf H2A.Z, H3K27ac and H3K18ac were all enriched around the subset of schizont-stage ATACseq peaks [41] that colocalise with enrichment of the invasion gene regulators PfAP2-I [8] and PfBDP1 [9] upstream of invasion genes (*n* = 151) (Fig. 8). The patterns of H3K27ac and H3K18ac enrichment were consistent with enrichment at the nucleosomes flanking the exposed DNA indicated by the ATACseq peak with bound PfAP2-I. However, PfBDP1 and Pf H2A.Z levels did not dip at the ATACseq peak summit (Fig. 8). Thus, for these genes it seems that Pf H2A.Z containing nucleosomes relatively depleted in the H3 acetylations were distributed across the summit of the ATACseq peak and some of these were bound by PfBDP1. Interestingly, PfBDP1 was also more enriched upstream than downstream of the ATACseq summit suggesting that PfBDP1 additionally binds nucleosomes upstream of the PfAP2-I binding site.

### Integrating ChIPseq and RNAseq to identify putative regulatory sequences

To investigate whether ICPs marked promoter or enhancer-like regulatory sequences, the ICPs were filtered by a set of stringent criteria to generate a set of sequences for further analysis (Additional file 16: Fig S11). For schizont stages, the 2645 ICPs were filtered for differential enrichment in H3K18ac and H3K27ac in schizonts compared to rings (*n* = 1371) and then filtered for the presence of an adjacent downstream gene within 2500 bp that was in the top quartile by expression in schizonts and upregulated at least threefold in schizonts compared to ring stages.

**Fig. 9:**
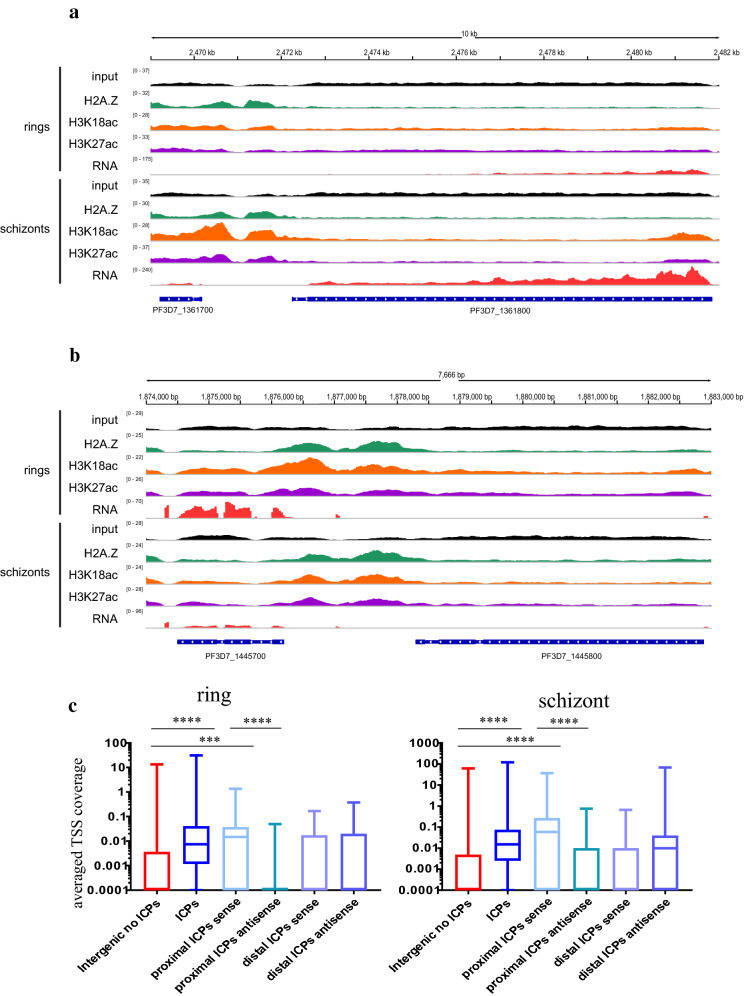
Tandem, intergenic colocalised peaks upstream of expressed genes have characteristics of regulatory sequences. Mapped reads normalised for mapped library size from concatenated replicates of input DNA and ChIP of Pf H2A.Z, H3K18ac and H3K27ac and from matched RNAseq for schizont and ring stages. Mapped reads are plotted over the preceding gene, the upstream intergenic sequence and a gene (**a**) in the forward orientation and expressed in schizont stages and (**b**) in the reverse orientation and expressed in ring stages. **c** As per Fig. 7b) Average, stranded, *P. falciparum* TSS tag coverage at 10 and 42 h post-invasion [42] at ICPs, all other intergenic regions and gene-proximal and distal ICPs from tandem pairs of ICPs. For this comparison the proximal and distal ICPs were defined as ± 50 bp from the colocalized H2A.Z peak summit. Kruskal–Wallis test showed significant variation across all categories in both rings and schizonts (*p* < 0.0001), shown are P values for post hoc comparisons (Dunn’s multiple comparisons test) **** *p* < 0.0001, ** *P* < 0.001

To identify potential promoter and enhancer-like sequence pairs marked by the ICPs, we further filtered the set of genes downstream of the filtered peaks for the presence of two sets of upstream, differentially enriched, colocalised ChIPseq peaks that were both closer to the downstream gene than any other gene. We hypothesised that the gene-proximal upstream ICP was a putative promoter whilst the gene-distal upstream ICP was a putative enhancer-like sequence. This generated a set of 30 differentially expressed genes that each had tandem upstream putative, regulatory sequences with differential enrichment of H3K18ac and H3K27ac and constitutive enrichment of Pf H2A.Z (Fig. 9a). A similar approach was used to identify 24 ring stage-expressed genes with tandem sets of upstream ICPs (Fig. 9b). However, the filtering for differential H3K18ac and H3K27ac enrichment was omitted for the ring-stage peaks because enrichment levels of these modifications are maintained throughout the IDC upstream of genes that are upregulated in ring stages (Fig. 5 gene set 2).

In both ring and schizont stages it was unlikely that the putative enhancer-like sequence exerted a positive regulatory effect upon the adjacent upstream gene because they were repressed or transcribed at low levels compared to the genes adjacent and downstream of the tandem ICPs (median, [IQR] rpkm of upstream/downstream genes in ring stages: 13 [3, 134]/606 [171, 978] and schizont stages: 41 [4, 224]/251 [134, 10537]; Wilcoxon test comparison between rpkm of upstream and downstream genes in rings *p* = 0.0002 and schizonts *p* = 0.005). In both stages, the putative promoters, but not enhancers, intersected with more TSS tags [42] than did intergenic sequences without ICPs (Kruskal–Wallis test both *p* < 0.0001, Dunn’s multiple comparisons test all *p* < 0.0003). The putative promoters also intersected more sense strand TSS tags than antisense (Dunn’s multiple comparisons test all *p* < 0.0001), but the putative enhancers did not (Fig. 9c). This showed that the putative promoters were driving sense transcription, consistent with promoter function, and that the putative enhancers did not have canonical promoter activity.

### Gene-proximal colocalised peaks of Pf H2A.Z, H3K18ac and H3K27ac mark sequences that have promoter activity

To investigate whether the putative promoter and enhancer pairs had regulatory activity, they were cloned upstream of a nanoluciferase reporter gene and used to transiently transfect *P. falciparum* along with a control plasmid expressing firefly luciferase*.* Transfected parasites were assayed for chemiluminescence normalised to transfected plasmid copy number and parasite genome number by quantitative PCR (qPCR) of matched DNA samples. We used qPCR for normalisation because firefly luciferase activity was too low for accurate detection. Luminescence was determined for parasites expressing nanoluciferase driven by (1) each of three schizont- and three ring-stage putative promoters, selected randomly from the 54 putative promoter sequences; (2) the hsp86 promoter as a positive control; (3) an AT content-matched *P. falciparum* intergenic sequence that lacked any chromatin modifications indicative of regulatory activity and that was located upstream of a non-expressed gene to control for non-sequence specific effects and (4) a no DNA control (Additional file 17: Table S5). Each of the putative promoter sequences also had the cognate, putative enhancer sequence cloned upstream and these were analysed for enhanced luminescence expression compared to plasmids containing the cognate promoter with a scrambled version of the putative enhancer from PF3D7_1362000 cloned upstream to control for non-sequence specific effects.

**Fig. 10:**
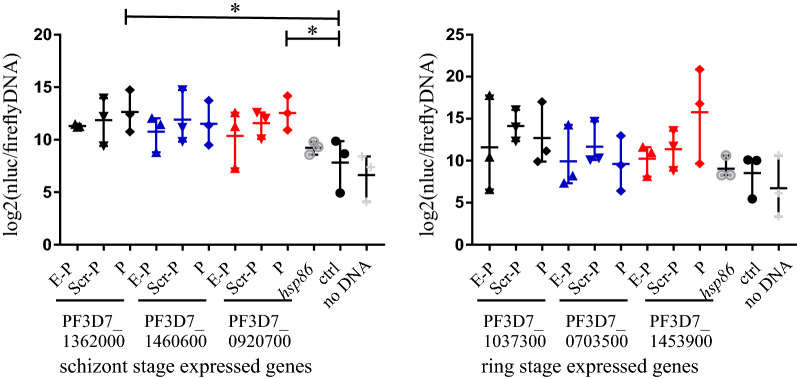
Transient transfections to detect whether intergenic colocalised peaks have gene regulatory activity. Shown are Log2 transformed nanoluciferase luminescence normalised to levels of firefly luciferase DNA and parasite genome copy number detected by qPCR. Each transfected plasmid encoded nanoluciferase with its transcription driven by upstream sequences consisting of: the putative enhancer upstream of the putative promoter (E-P); a scrambled version of the PF3D7_1362000 putative enhancer upstream of the putative promoter (Scr-P); the putative promoter alone (P); the *hsp86* promoter (*hsp86*); an AT-matched intergenic control sequence that had no regulatory effect on neighbouring genes during the asexual lifecycle (ctrl) and a mock transfection control (no DNA). ANOVA with post hoc Sidak’s multiple comparisons test of the AT-matched control sequence to each of the putative promoter-alone constructs and the hsp86 promoter alone. *p < 0.05. Bars are the means, whiskers are the ranges

Two of the schizont-stage putative promoters had greater promoter activity than the AT content-matched control sequence (ANOVA *p* = 0.0205, Sidak’s multiple comparisons test PF3D7_1362000 *p* = 0.0196, PF3D7_0920700 *p* = 0.0228) and the trend to greater activity approached significance for the third schizont-stage putative promoter (PF3D7_1460600 *p* = 0.0908), but none of the three ring-stage putative promoters had greater activity than the AT content-matched control sequence (Fig. 10). Curiously the hsp86 control promoter sequence had no greater activity than the AT content-matched control sequence, indicating that the control sequence itself had moderate promoter activity and that the gene-proximal ICPs had identified strong schizont-stage promoters. None of the three ring stage nor three schizont-stage putative enhancers affected activity of the cognate, putative promoter. We concluded that the distal ICPs in the paired sets of ICPs did not correspond to enhancer-like elements.

## Discussion

**Fig. 11:**
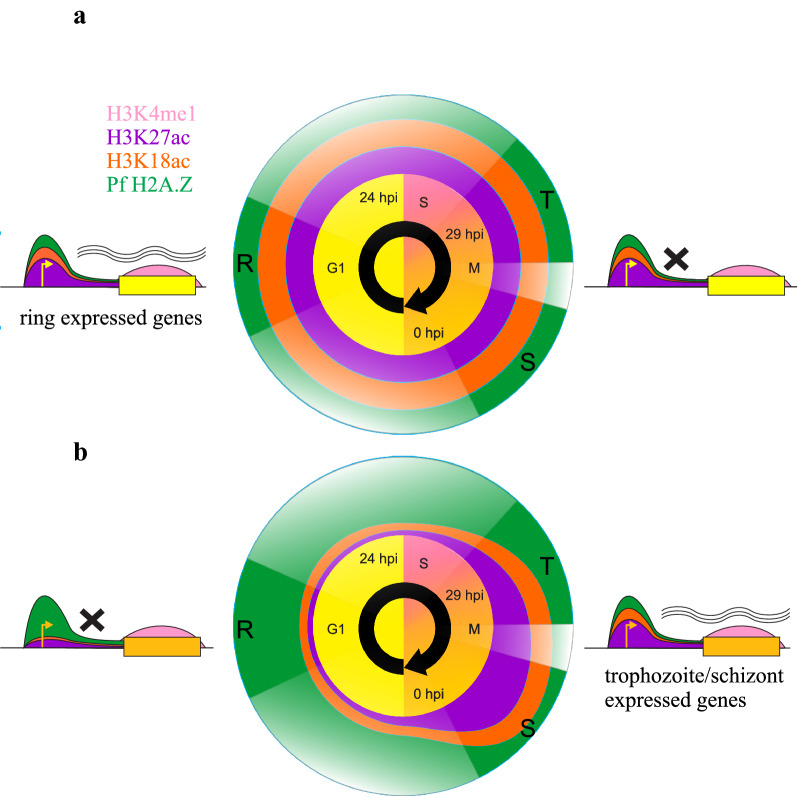
Proposed model of Pf H2A.Z, H3K4me1, H3K18ac and H3K27ac dynamics across transcriptional units throughout the intra-erythrocytic developmental cycle. Enrichment patterns of Pf H2A.Z (green), H3K18ac (orange), H3K27ac (purple) and H3K4me1 (pink) throughout the IDC at genes expressed in ring stages or in trophozoites/schizonts. The positions of enrichment of Pf H2A.Z and the H3 modifications are indicated on the models of genes expressed in ring (**a**) stages during G1 (yellow genes) or (**b**) in trophozoites and schizonts during asynchronous S phase and mitosis (M) (tan genes). The temporal profiles of abundance of Pf H2A.Z, H3K18ac and H3K27ac at the promoters of genes expressed in (**a**) ring stages or (**b**) trophozoites and schizonts are indicated as coloured concentric circles interpolated onto the full cell cycle. Distortions in the concentric circles indicate relative abundance of the histones (modifications) in those periods. The model was interpolated from the ChIPseq experiments conducted on parasites spanning 6-h windows indicated by solid-coloured wedges at 8–14 hpi, rings (R), 30–36 hpi trophozoites (T) and 38–44 hpi schizonts (S). The profiles in the transparent wedges were inferred but are unknown, particularly whether abundance of the H3 acetylations decreased at promoters of ring stage-expressed genes between 14 and 30 hpi

Throughout the *P. falciparum* intra-erythrocytic developmental cycle Pf H2A.Z, H3K18ac and H3K27ac were enriched in intergenic sequence. A model is proposed in Fig. 11 that describes the patterns of enrichment of Pf H2A.Z, H3K18ac, H3K27ac and H3K4me1 throughout the intra-erythrocytic developmental cycle and their associations with gene expression. As previously reported, the intergenic enrichment of Pf H2A.Z correlated with steady-state expression levels, but Pf H2A.Z was not dynamically enriched at expressed genes [59]. In other eukaryotes, H2A.Z is required to mark promoters that are subsequently activated by other chromatin dynamics [63, 64]. H3K18ac and H3K27ac were also enriched at, and upstream of, the TSS but their intergenic enrichment dynamically correlated with gene expression. H3K18ac and H3K27ac thus marked active promoters and possibly additional regulatory elements, consistent with evidence from other eukaryotes [10–12, 20, 23–25, 65].

H3K18ac and H3K27ac appeared to accumulate at intergenic sites during asynchronous S phase and mitosis and were removed during the subsequent G1 phase following expression of the cognate, downstream gene (Fig. 11). Thus, these marks at a promoter preceded expression of the cognate gene. Increased accessibility of promoters has been previously noted to also precede their activity [40]. Maximum abundance of H3K27ac during mitosis and enrichment of H3K27ac during mitosis upstream of genes expressed at any stage during the following cell cycle has also been described in human cells [66]. H3K27ac enrichment at human regulatory sequences in mitosis is also predictive of a transcriptional burst in human cells at the mitosis/G1 transition, leading to the proposition that H3K27ac may be important for cell cycle progression [67].

We had observed this pattern of H3K27ac enrichment before in a limited set of *P. falciparum* genes including active *var* genes [37]. The genome-wide pattern revealed here by ChIPseq does not exclude a role for H3K18ac and H3K27ac in epigenetic memory for *var* gene regulation, but the apparent persistence of these marks throughout the cell cycle is more probably a consequence of the genome-wide timing of deposition and removal of these histone modifications.

The low complexity of the *P. falciparum* intergenic sequence with approximately 90% AT content has hindered attempts to identify gene regulatory sequences. Studies of *P. falciparum* genes using episomes with various truncations of upstream regions characterised core promoters around the TSS, but also identified additional sequences starting between 300 and 1400 bp upstream of the TSSs that were required for efficient promoter activity [68–73], that conferred stage-specific expression [72, 73], that bound nuclear *trans* factors [69, 71] and that were functional regardless of orientation, a classical eukaryotic enhancer characteristic [69–72]. These repeated demonstrations of *cis* regulatory elements that act upon a core promoter are convincing evidence of enhancer or UAS-like elements that bind specific transcription factors to regulate gene expression in *P. falciparum*.

As the enrichment levels of intergenic H3K18ac and H3K27ac were highly correlated and dynamically associated with downstream gene expression, we attempted to use these modifications to define potential regulatory sequences in the intergenic regions upstream of genes. This approach could not detect enhancer-like sequences that were distant from genes or in gene-bodies, although Hi-C studies suggest the former are unlikely to function in asexual blood stage *P. falciparum* [45, 46]. Throughout eukaryotes H2A.Z is also enriched around regulatory sequences so colocalised peaks of Pf H2A.Z, H3K18ac and H3K27ac (ICPs) were analysed for characteristics of regulatory sequences. The coverage of published TSSs [42] by the ICPs was consistent with them marking regulatory sequences. In *P. falciparum,* intergenic nucleosomal occupancy peaks at the well positioned + 1 nucleosome that overlies the TSS, this is immediately downstream of a short nucleosome depleted region that is itself downstream of a region of above average occupancy of poorly positioned nucleosomes [43]. The ICPs overlaid a sharply defined MNaseSeq peak representing a nucleosome with a position conserved between the parasites analysed in these orthogonal studies, presumably equivalent to the strongly positioned + 1 nucleosome at the TSS [43].

The observed stage-dependent proximity of ICPs to ATACseq peaks and the patterns of histone modification enrichment at ATACseq peaks suggest that these ICPs demarcate regulatory sequences that bind transcription factors. The example of the AP2-I/PfBDP1 association with ICPs clearly demonstrated enrichment of these histone modifications at nucleosomes flanking the bound transcription factor at the ATACseq peaks. PfBDP1 was recently shown to have promiscuous affinity for the Pf H2A.Z acetylations [74] that alone, or in combination with other, untested histone acetylations, may be actually binding PfBDP1 explaining the relative depletion of H3K18ac and H3K27ac at the summits of the PfBDP1/AP2-I peaks.

We further filtered ICPs by successive, stringent criteria to generate a small set of putative, proximal promoter and distal enhancer-like regulatory sequence elements. Our attempt to infer the presence of regulatory sequences from the presence of expression-associated ICPs was clearly corroborated by the promoter activity of sequences underlying the schizont-stage, gene-proximal ICPs in transient transfection experiments. However, the ring stage sequences and the schizont-stage putative enhancers did not have regulatory effects on the episomal reporter. We concluded that the ICPs we analysed did not define sequences with enhancer function that was detectable using our transient transfection system. It is probable that ATACseq datasets contain the identities of enhancer or UAS-like elements in *P. falciparum*. However, the large number of ATACseq peaks detected in those studies [40, 41] means that intersection with other orthogonal datasets is required to filter false positives and identify the enigmatic, regulatory elements that lie outside the core promoter. The ChIPseq datasets we provide here would be a valuable contribution to such a meta-analysis.

The intergenic depletion and coding sequence enrichment of H3K4me1 was consistent with previous reports from *P. falciparum* [39], yeast [13, 75], and humans [18]. However H3K4me1 was not enriched upstream of the TSS as it is in yeast [13, 75, 76], nor at nucleosomes flanking the TSS and at enhancers as it is in humans [16, 18, 21]. Intergenic levels of H3K4me1 inversely correlated with downstream gene expression consistent with the association in yeast between H3K4me1 around the TSS and gene repression [65]. However, upstream H3K4me1 depletion did not vary with dynamic gene expression indicating that H3K4me1 was not marking regulatory sequences. In *P. falciparum* H3K4me3 is largely not dynamically enriched at active promoters, but instead levels increase throughout the lifecycle with the greatest intergenic enrichment in trophozoites and schizonts at the most highly expressed genes [38]. This pattern inversely mirrors the profile of intergenic H3K4me1, so we concluded that the accentuated depletion of H3K4me1 at highly expressed promoters was likely a consequence of additional methylation of H3K4 to H3K4me3 and that H3K4me1 was not specifically a feature of regulatory sequences in *P. falciparum*.

A recent study reported that intergenic enrichment of H3K4me1 in *P. falciparum* marked regulatory regions [48]. However, the intergenic sequences that were enriched in H3K4me1 in that study had chromatin characteristics more like coding sequence than regulatory sequence and they did not lie upstream of highly expressed genes, making it unlikely that they had enhancer function. These sequences may be analogous to the GC-rich (upstream) uORFs, previously implicated in post-transcriptional regulation of *var* genes [77, 78], although none of the H3K4me1 peaks lay upstream of a *var* gene.

## Conclusions

In conclusion, we show that the histone modifications H3K18ac and H3K27ac mark promoters preceding and during gene expression, that they demarcate regulatory sequences defined by ATACseq and that together with colocalised *Pf* H2AZ they identify schizont stage sequences with promoter activity. In contrast, H3K4me1 did not appear to be associated with intergenic sequences exerting a regulatory effect, but the small number of intergenic H3K4me1 peaks identified did have a curious chromatin and GC composition suggestive of coding sequence, which may warrant further investigation. These findings contribute to the understanding of chromatin structure at the poorly defined gene regulatory elements of *P. falciparum* and how the contribution of histone modifications to gene regulation may be influenced by the *P. falciparum* cell cycle.

## Materials and methods

### Parasite culture

The *P. falciparum* 3D7 clone was cultured [79] using a modified method. Briefly, parasites were grown in RPMI–HEPES supplemented with 0.5% AlbuMAX® II Lipid-Rich BSA (Life technologies, 11,021), 0.2% sodium bicarbonate and O + red blood cells at 3% hematocrit. Parasites were incubated at 37 °C in 5% CO_2_, 1% O_2_ and 94% N_2_. Ring-stage parasites were synchronised to an approximately 4-h window by two incubations with 5% D-sorbitol [80] separated by a 12-h interval.

### Antibodies

Primary antibodies employed in dot blot, immunofluorescence assay, western blot and ChIP assays in this study were rabbit anti-Pf H2A.Z and pre-immune rabbit serum [50], rabbit anti-H3 (Abcam, Ab1791), rabbit anti-H3K4me1 (Abcam, Ab8895), rabbit anti-H3K27ac (Abcam, Ab4729), rabbit anti-H3K18ac (Abcam, Ab1191) and rabbit control IgG (Abcam, Ab46540). Secondary antibody used for dot blot and western blot was goat anti-rabbit IgG antibody conjugated with horseradish peroxidase (HRP) (Invitrogen, A16110). Secondary antibody for immunofluorescence assay was Alexa Fluor® 488 anti-rabbit antibody (life technologies).

### Western blots

Parasites at 5–10% parasitemia were harvested at 8-h intervals, erythrocytes were lysed in 0.075% saponin and parasite pellets then dissolved in 2 × reducing Laemmli buffer (125 mM Tris HCl pH 6.8, 20% glycerol, 4% SDS, 0.005% bromophenol blue, 5% beta-mercaptoethanol) at a concentration of 5 × 10^6^ infected erythrocytes/μl. Reduced parasite lysates were denatured at 95 °C for 5 min and approximately 1 × 10^7^ infected erythrocytes were separated by SDS-PAGE on NuPAGE® Novex® 4–12% Bis–Tris protein gel (Life technologies, NP0323BOX) in 1 × NuPAGE® MES SDS running buffer (Life technologies, NP0002) at 150 V for 70 min. Proteins were transferred to an Amersham HYBOND-ECL membrane (GE Healthcare, 45-000-929) in cold transfer buffer (192 mM glycine, 25 mM Tris, 3.5 mM SDS, 20% methanol) at 400 mA for 1 h and successful transfer was verified by Ponceau S (0.1% Ponceau S, 5% acetic acid) staining. The membranes were incubated with primary and secondary antibodies and bound antibodies were detected using Immobilon Western Chemiluminescent HRP Substrate (Merck Millipore, WBKLS0500).

### Dot blot

Custom-made, biotinylated *P. falciparum* peptides PfH3 unmodified 1 (residues 1–21), PfH3 unmodified 2 (residues 21–43), PfH3 unmodified 3 (residues 47–65), PfH3K4me1 (residues 1-21), PfH3K9ac, PfH3K14ac, PfH3K18ac, PfH3K27ac, PfH3K23,27ac and PfH3K56ac and biotinylated human peptides HuH4 (residues 1–21) (Epigentek, R-1007-100), HuH4K20me1 (Epigentek, R-1054-100), HuH4K20me3 (Epigentek, R-1056-100) and nonbiotinylated human peptides HuH3K4me3 (Abcam, Ab1342), HuH3K36me3 (Abcam, Ab1785) and HuH3K9me3 (Abcam, Ab1773) were used for testing antibody specificity. All *P. falciparum* peptides and nonbiotinylated human peptides were used at 0.25 mg/ml except for HuH4K20me1 and HuH4K20me3 which were used at 0.07 mg/ml while HuH4 was 0.065 mg/ml. All peptides were then tested at further dilutions of 1:4, 1:8, 1:16 and 1:32. One microlitre of each dilution was loaded on an Amersham Hybond^TM^ HYBOND-ECL membrane (GETM Healthcare, 45-000-929). One membrane was blocked with 5% w/v skim milk powder in TBS-T, washed, incubated with streptavidin–HRP diluted 1:50,000 in TBS-T, washed, and the membrane bound streptavidin–HRP was detected using Immobilon Western Chemiluminescent HRP Substrate (Merck Millipore, WBKLS0500). The other membranes were blocked with 5% w/v skim milk powder in in TBS-T, each was incubated with a primary antibody, washed, incubated with secondary antibody conjugated to HRP, washed again then bound antibodies were detected using Immobilon Western Chemiluminescent HRP Substrate (Merck Millipore, WBKLS0500).

### Immunofluorescence analysis

Smears of asynchronous parasite cultures were prepared on glass slides and air-dried overnight. Cells were fixed and permeabilised by a mixture of ice cold 10% methanol/90% acetone for 5 min at room temperature and air-dried. Cells were rehydrated in PBS, stained with primary antibodies, washed with PBS, stained with secondary antibody and DAPI (Sigma-Aldrich, D9542), washed, mounted in ProLong® Gold Antifade Reagent (Life technologies, P36934) and left to cure overnight. Imaging was performed using a Zeiss Fluorescence Upright Microscope (ZEISS Axioskop 2 mot plus) with the 100 × oil objective.

### Cross-linked chromatin immunoprecipitation

Chromatin was isolated from ring-stage (8–14 h post-invasion), trophozoite-stage (30–36 hpi) and schizont-stage (38–44 hpi) parasites as previously described [50]. Briefly, infected erythrocytes were cross-linked with 1% formaldehyde at 37 °C for 10 min and quenched with 125 mM glycine. Erythrocytes were lysed with saponin and parasites were incubated for 30 min on ice in 10 mM HEPES pH 8.0, 10 mM KCl, 0.1 mM EDTA pH 8.0, 0.1 mM EGTA pH 8.0, 1 mM DL-dithiothreitol (DTT) and 1 × EDTA free protease inhibitors. IGEPAL® CA-630 (Sigma-aldrich, I8896) was then added to a final concentration of 0.25% before lysing parasites with a Dounce tissue grinder (Sigma-aldrich, D8938-1SET, Pestle B). The nuclei were pelleted at 21,000×*g* for 10 min at 4 °C and nuclei from 2 × 10^9^ ring-stage parasites, 1 × 10^9^ trophozoite-stage parasites or 4 × 10^8^ schizont-stage parasites were resuspended in 200 μl SDS lysis buffer (1% SDS, 10 mM EDTA, 50 mM Tris pH 8.1, 1 × EDTA free protease inhibitors). Chromatin was sheared into 200–600 bp fragments by sonication in a Bioruptor UCD-200 (Diagenode) for 28 min (High, 30 s on, 30 s off) and diluted 1:10 in ChIP dilution buffer (0.01% SDS, 1.1% Triton X-100, 1.2 mM EDTA, 16.7 mM Tris–HCl pH 8.1, 150 mM NaCl).

For each immunoprecipitation, diluted chromatin was pre-cleared using protein A/G beads (GE Healthcare Life Sciences, 17-5280-01&17-0618-01) at a beads-to-chromatin volume ratio of 1:200 for 1 h at 4 °C. Approximately 470 μl pre-cleared chromatin from 5 × 10^8^ ring-stage or 2.5 × 10^8^ trophozoite-stage or 1 × 10^8^ schizont-stage parasites were immunoprecipitated overnight at 4 °C with 8 μg antibody and 30 μl prewashed protein A/G beads. 1/10th of the volume of immunoprecipitated chromatin was also incubated overnight at 4 °C for use as the input control. Beads were washed once with low-salt immune complex wash buffer (0.1% SDS, 1% Triton X-100, 2 mM EDTA, 20 mM Tris–HCl pH 8.1, 150 mM NaCl), once with high-salt immune complex wash buffer (0.1% SDS, 1% Triton X-100, 2 mM EDTA, 20 mM Tris–HCl pH 8.1, 500 mM NaCl), once with LiCl immune complex wash buffer (0.25 M LiCl, 1% NP-40, 1% deoxycholate, 1 mM EDTA, 10 mM Tris–HCl pH 8.1) and twice with TE buffer (10 mM Tris–HCl pH 8.1, 1 mM EDTA pH 8.0) and co-immune-precipitated DNA was then eluted twice with freshly made elution buffer (1% SDS, 0.1 M NaHCO_3_). Reversal of cross-linking was performed by incubating the eluate at 45 °C overnight with 500 mM NaCl. DNA was purified using the MinElute PCR purification kit (Qiagen, cat 28,006). All ChIP experiments were performed in three biological replicates.

### ChIPed DNA library preparation for sequencing

ChIPed DNA libraries were prepared using the NEBNext® ultra DNA library prep kit for Illumina® (New England BioLabs, E7370). 6 ng input or chromatin-immunoprecipitated DNA was used and adaptor-ligated DNA was purified without size selection using Agencourt AMPure XP beads (Beckman coulter, A63881). The PCR amplification step was performed using 1 unit of high-fidelity KAPA HiFi DNA polymerase (KAPA biosystems, KK2101), 0.3 mM dNTPs, 0.5 μM NEBNext Universal PCR Primers for Illumina and 0.5 μM NEBNext Index Primer for Illumina (NEBNext® Multiplex Oligos for Illumina® Index Primers Set 1 (E7335) and Set 2 (E7500)) in 1 × KAPA HiFi buffer containing tetramethylammonium chloride for 12 PCR cycles [81]. The PCR conditions were 98 °C for 2 min for the initial denaturation followed by 12 cycles of 98 °C for 15 s and 65 °C for 2 min and a final extension at 65 °C for 5 min. Library clean-up was performed using 1 × AMPure beads and DNA libraries were eluted with 10 mM Tris–HCl pH 7.5.

### RNA library preparation for sequencing

Stage-matched RNA from at least two biological replicates was extracted, gDNA digested and RNASeq libraries constructed as described previously [82]. Briefly, the aqueous phase from cells dissolved in TRIzol® reagent (Life Technologies, 15596018) was diluted in 70% ethanol and purified using RNeasy Mini columns (Qiagen, cat 74104). DNase digestion with the RNase-Free DNase Set (Qiagen, cat 79254) was repeated until qPCR of the DNased RNA gave a Ct greater than 34 for the *P. falciparum hsp70* gene. Total RNA (2.5 μg) was depleted of haemoglobin mRNA using the GLOBINclear™ Kit (Life Technologies, AM1980) and mRNA then purified using magnetic oligo d(T) beads (NEBNext® Poly(A) mRNA Magnetic Isolation Module, New England BioLabs, E7490S). Indexed mRNA libraries were prepared using the NEBNext® Ultra™ Directional RNA Library Prep Kit for Illumina® (New England BioLabs, E7420S). PCR enrichment was performed as per the ChIPseq method described above. The PCR conditions were 98 °C for 1 min for the initial denaturation followed by 12 cycles of 98 °C for 10 s and 65 °C for 1 min and a final extension at 65 °C for 5 min. Library clean-up was performed using 0.8 × AMPure beads and RNA libraries were eluted with nuclease-free water.

### High-throughput sequencing

Input and chromatin-immunoprecipitated DNA libraries and mRNA libraries were subjected to quantification and quality check for purity and fragment size range using the Agilent Bioanalyzer and Qubit Fluorometer analyses prior to sequencing by the HiSeq2500 (Illumina) at the Melbourne Translational Genomics Platform. 126-bp paired-end reads were generated.

### Bioinformatic analyses

Bioinformatic analysis was performed on Ubuntu 14.04.5 LTS (GNU/Linux 3.13.0–105-generic x86_64). Quality control of all raw sequencing data was performed using FastQC (version 0.11.2, https://www.bioinformatics.babraham.ac.uk/projects/fastqc/). Adaptor sequences were trimmed and reads were quality filtered using Trim Galore! (version 0.3.7, www.bioinformatics.babraham.ac.uk/projects/trim_galore/). ChIP-seq and RNA-seq reads from biological replicates from each stage mapped to *P. falciparum* 3D7 genome were visualised using IGV [83]. Data range for each track was normalised to the corresponding library size. Replicate reproducibility was checked using MultiBamSummary, PlotCorrelation and PlotPCA tools from DeepTools [84] with default settings.

### RNAseq

RNA library sequencing reads were mapped to the *P. falciparum* 3D7 genome assembly using Tophat2 [85]. The resulting BAM files of biological replicates were concatenated using Samtools [86]. Transcript assembly was performed using Cufflinks on concatenated BAM files [87]. Transcripts or genes were ranked by isoform-level or gene-level expression values presented as fragments per kilobase of exon per million reads mapped (FPKM) in each parasite stage during the IDC. In each stage, genes with Cufflinks IDs were divided into four groups by expression levels, i.e. three terciles and silent genes. Any Cufflinks gene ID that could not be assigned to any *P. falciparum* 3D7 gene or was assigned to multiple genes was omitted from the analysis. Differential gene expression analysis between any two stages of the IDC was performed using Cuffdiff [87].

### ChIPseq

Input DNA and chromatin-immunoprecipitated DNA library sequencing reads were mapped to the *P. falciparum* 3D7 genome (PlasmoDB v12) using Subread [88]. The resulting BAM files of biological replicates were concatenated using Samtools [86]. H2A.Z, H3K18ac, H3K27ac and H3K4me1 peaks from each stage were called from concatenated BAM files using MACS2 (*q* = 0.01) [89]. NGSplot [90] was used to plot log2ChIP/(input or H3 ChIP) across specified genomic regions as averaged line plots and heatmaps.

Schizont and ring-specific candidate enhancers and promoters and their cognate genes were identified using BEDTools [91]. Briefly, to identify schizont-specific candidate regulatory elements, schizont-stage colocalised, intergenic H2A.Z, H3K18ac and H3K27ac peaks were detected using ‘bedtools intersect’ and peak boundaries were defined using H2A.Z peaks. H3K18ac and H3K27ac ChIP-seq peaks that were differentially enriched in schizonts versus rings were identified using csaw FDR < 0.05 [92] and intersected with the schizont-stage colocalised, intergenic H2A.Z, H3K18ac and H3K27ac peaks using ‘bedtools intersect’. Peaks were then filtered for the presence of a gene within 2500 bp downstream that was expressed at least threefold more in schizont stages than in ring stages and that were in the top quartile by schizont-stage expression (gene set 1 from Fig. 5) using ‘bedtools window’. Finally, the closest differentially expressed genes to two upstream colocalised, intergenic, H3K18ac and H3K27ac peaks with differential stage enrichment were identified using ‘bedtools closest’. The closer peak to the gene was the putative promoter while the further peak was the putative enhancer.

### Comparisons with published datasets: nucleosome occupancy, transcription start sites, DNA accessibility, PfBDP1 and PfAP2-I

*P. falciparum* MNase-seq read coverage normalised per site in BEDGRAPH format at time points 15 h post-invasion and 40 h post-invasion was retrieved from the Gene Expression Omnibus GSE66185 [43]. Coordinates of total 5590 genes from *P. falciparum* 3D7 v12 were retrieved from PlasmoDB (https://plasmodb.org). Coordinates of total 5567 intergenic regions were obtained using “bedtools complement”. The average score of BEDGRAPH records that intersected with *P. falciparum* intergenic regions and colocalised H2A.Z, H3K18ac and H3K27ac peaks was determined using “bedtools map”. Mann–Whitney U test was performed using GraphPad Prism version 6.

Stranded *P. falciparum* TSS tag coverage at 10 and 42 h post-invasion were retrieved from the Gene Expression Omnibus GSE68982 [42]. 2196 ring- and 2943 schizont-stage intergenic, colocalised peaks were identified as described above. For this comparison the peaks were defined as ± 50 bp from the colocalised H2A.Z peak summit. Mann Whitney U test or Kruskal–Wallis test and Dunn’s multiple comparisons test were performed using GraphPad Prism version 6.

ATACseq data from *P. falciparum*-3D7-t40 replicate 1 GSM2789028 was mapped to the *P. falciparum* 3D7 strain genome release 12 version 3 (PlasmoDB) using BWA-MEM [93] as per [41] and peaks were called using MACS2 [89]. The same schizont-stage ATACseq data normalised to gDNA was converted from the supplied bedgraph file ncbi accession GSM2789028 to bigwig format using bedGraphtoBigWig (UCSC) [94] in bioconda [95]. The resulting bigwig file was used in subsequent graphical visualisations in deepTools [84].

The schizont-stage MNase-seq T40A sample from SRX885818: GSM1616491: comprised 5 paired-end fastq libraries SRR1813391 to SRR1813395 [43]. Adapters and low-quality ends (phred < 20) were trimmed using trim_galore (cutadapt) (version 0.3.7, www.bioinformatics.babraham.ac.uk/projects/trim_galore/) and reads then mapped to the *P. falciparum* 3D7 strain genome release 12 version 3 (PlasmoDB) by BWA-MEM v 0.7.17 [93] and filtered using samtools [86] for alignments with quality > 30 and to remove alignments that were a PCR or optical duplicate or were a secondary or supplementary alignment (flags 256, 1024, 2048) as per [43]. The bam files were concatenated and used to make bigwig files for analysis in deeptools [84].

The schizont-stage AP2I ChIPseq and input data [8] from GEO: GSE80293 comprising 3 replicate schizont-stage AP2I ChIP and 3 matched input fastq files were trimmed of adapters and low-quality ends (phred < 20) using trim_galore (cutadapt) (version 0.3.7, www.bioinformatics.babraham.ac.uk/projects/trim_galore/) and then reads were mapped to the *P. falciparum* 3D7 strain genome release 12 version 3 (https://plasmodb.org) by BWA-MEM v 0.7.17 [93] and filtered using samtools [86] for alignments with quality > 30 and to remove alignments that were a PCR or optical duplicate or were a secondary or supplementary alignment (flags 256, 1024, 2048) as per [8]. The bam files were concatenated and used to make bigwig files for analysis in deeptools [84]. The duplicate schizont-stage PfBDP1 ChIPseq and input data were processed as per [9] to generate bam files and MACS2 [89] called peaks.

In order to compare ICP peaks with chromatin accessibility across asexual stages, ATACseq data were acquired and mean peak positions derived from the start and end of the reported peaks [40]. The distance between each H2AZ peak summit and the nearest ATAC-seq peak was calculated for each of the ATAC-seq stages reported. These distances were plotted for ICP versus non-ICP Pf H2AZ peak summits [40] (Fig. 7c), and compared pairwise using Welch's t-test with Bonferroni correction.

## Transient transfections

### Plasmid constructs

To create the control plasmids for nanoluciferase expression, the sequence encoding nanoluciferase was cloned into the pPf86 plasmid [96] using the *Nco*I and *Xba*I restriction sites to create the plasmid pNluc. An AT content-matched *P. falciparum* intergenic sequence that lacked any chromatin modifications indicative of regulatory activity and that was located upstream of a non-expressed gene was used to control for non-sequence specific effects (Additional file 17: Table S5). The control sequence was amplified from *P. falciparum* 3D7 genomic DNA and cloned into *Mlu*I and *Xho*I restriction sites in pNluc, directly upstream of the hsp86 promoter to generate the pNR3 control plasmid.

The putative promoter and enhancer sequences were amplified from *P. falciparum* 3D7 genomic DNA (Additional file 17: Table S5). Candidate promoter sequences were cloned into *Xho*I and *Nco*I restriction sites of the pNluc plasmid using Gibson assembly. The corresponding enhancer sequences were cloned into these plasmids using the *Mlu*I and *Xho*I restriction sites. In parallel, the scrambled enhancer sequence was designed by scrambling the putative enhancer sequence for PF3D7_1362000 using the Sequence Manipulation Suite (https://www.bioinformatics.org/sms2/shuffle_dna.html), and synthesising the sequence with GeneArt (Life Technologies), and also cloned into the *Mlu*I and *Xho*I restriction sites of the promoter-containing pNluc constructs. The promoter-only constructs were made by digesting promoter-containing constructs using *Mlu*I and *Xho*I restriction enzymes, removing overhangs using Klenow fragment and ligating to form the final plasmid.

### Transient transfections

5 µg of plasmids containing test sequences driving Nluc, along with 15 µg of pPf86 [96] were ethanol precipitated and resuspended in 10 µL of sterile TE buffer. Schizont-stage parasites were purified from a 67% (v/v) Percoll cushion and resuspended in 100 µL supplemented Nucleofector™ solution (Lonza) containing the plasmid DNA. The parasites were transfected with the Amaxa^TM^ 4D-Nucleofector™ System (Lonza), using program FP158. The transfected parasites were immediately cultured in medium containing fresh erythrocytes and harvested 6–8 days after transfection. The parasites transfected with putative ring-stage regulatory elements were harvested at ring stage and the parasites transfected with putative schizont-stage regulatory elements were harvested at schizont stage.

### Luciferase measurement

Parasites were treated with 0.1% (w/v) saponin in PBS and washed in PBS. Freed parasites were added to white 96-well plates and treated with NanoDLR™ Stop & Glo reagent, to measure nanoluciferase activity. In addition, the levels of firefly luciferase DNA and the number of parasite genomes were measured by quantitative PCR using SYBR™ Green PCR Master Mix (ThermoFisher). The level of nanoluciferase activity was calculated relative to the amount of firefly luciferase DNA per parasite genome to determine the expression of nanoluciferase per plasmid per parasite for all transfections.

## Supplementary information

**Additional file 1: Fig S1.** Specificity of ChIPseq antibodies for *P. falciparum* histones. Western blot of *P. falciparum* whole-cell lysate probed with the antibodies used in this study and with antibody to H4K12ac as an additional control showing the presence of multiple histones on the western blot.

**Additional file 2: Fig S2.** Specificity of ChIPseq antibodies for *P. falciparum* histone modifications. Dot blot of biotinylated histone peptides probed with rabbit antibody to a) H3K18ac (Abcam, Ab1191), b) rabbit antibody to H3K27ac (Abcam, Ab4729), c and f) rabbit antibody to H3K4me1(Abcam, Ab8895) and d and e) streptavidin as a loading control. Peptides indicated on the left were diluted from right to left as shown above the figure. Starting concentrations (neat) were 0.25 mg/ml for all *P. falciparum* (Pf) peptides and Human (Hu) H3K4me3, H3K36me3 and H3K9me3 peptides; 0.07 mg/ml for Human H4K20me1 and H4K20me3; 0.065 mg/ml for Human H4. Human H3K4me3, H3K36me3 and H3K9me3 were not biotinylated and their loading was confirmed by ponceau red staining shown for neat peptides in lanes to the left. *P. falciparum* H3 unmodified 1, 2 and 3 peptides represented residues 1–21, 21–43 and 47–65, respectively. A minor cross reaction of anti-H3K18ac antibody with a *P. falciparum* histone H3 unmodified peptide (residues 47–65) was observed, but the antibody was at least 32-fold more sensitive for H3K18ac than H3 (residues 47–65). The rabbit anti-H3K27ac antibody strongly bound both the *P. falciparum* H3K27ac peptide and the H3K23acK27ac peptide. The anti-H3K27ac antibody also bound the *P. falciparum* H3K9ac peptide but by densitometry it generated no more than 1/32 of the signal of H3K9ac.

**Additional file 3: Table S1.** Quality summary of ChIPseq data.

**Additional file 4: Table S2.** Quality summary of RNAseq data.

**Additional file 5: Fig S3.** Reproducibility of ChIPseq. PCA plots and associated scree plots for all ChIPseq and input replicates for ring stages, trophozoite stages and schizont stages.

**Additional file 6: Fig S4.** Individual sequencing tracks. Read coverage for ChIP, input and RNA sequencing replicates across 78 kb within chromosome 14.

**Additional file 7: Fig S5.** Correlations between expression level and histones and their modifications across transcriptional units. Log2 (ChIP/input) plotted from 2500 bp upstream (−2500) to 2500 bp downstream (2500) of the transcriptional start (TSS) and stop (TES) sites for 7612 ring stage transcripts and 7711 trophozoite stage transcripts assembled by Cufflinks ranked in descending order by transcript abundance (fpkm). The Spearman r correlation value (*r*) for the upstream and downstream enrichment of each histone or histone modification is indicated below the plots.

**Additional file 8: Fig S6.** Average enrichment profiles of histones and their modifications across genes binned by expression level. The average profile of H2A.Z, H3K18ac, H3K27ac, H3K4me1 and H3 over the coding sequences ± 2500 bp for genes grouped into terciles by expression level (top green, middle orange, bottom purple) and for silent genes (pink). The enrichment profile of each chromatin mark was presented as the log2 ratio over the input control. In rings, trophozoites and schizonts gene expression categories were, respectively, the top (*n* = 1636, 1603, 1613), middle (*n* = 1495, 1539, 1519) and bottom (*n* = 1580, 1356, 1386) terciles, and silent genes (n*n*= 476, 530, 602). 5′End: the start codon of a gene; 3′End: the stop codon of a gene.

**Additional file 9: Fig S7.** A) Average enrichment profiles of chromatin features and RNA levels from this study across the summits ± 1.8 kb of intergenic, peaks of H3K4me1 ChIP from this study relative to H3K4me3 from [38] B) Average enrichment profiles of chromatin features and RNA levels from this study across the intergenic peaks of H3K4me1 identified by Ubhe et al [48]. Average profile plots ± SE covering 1.5 kb up and downstream of published, “strong” H3K4me1 intergenic peaks longer than 1 bp (*n* = 259) [48] oriented according to the closest downstream gene. Top panel is the log2 ratio of ChIP over input average enrichment profiles of H3K4me1, H3K27ac, H3K18ac, H3 and Pf H2A.Z, middle panel is GC content and ATACseq normalised to gDNA and the bottom panel is RNA separated by strand. C) The number of intergenic peaks and their intersections from the H3K4me1/input from this study, the H3K4me1 from this study over the H3K4me3 from [38], and the H3K4me1 enriched intergenic regions from [48]. D) Spearman correlations for the single schizont H3K4me1 ChIP sample and matched input (Ubhe) analysed in [48] and for our two schizont replicates (S1 and S2) of H3K4me1 ChIP and matched input.

**Additional file 10: Table S3.** Number of highly expressed genes differentially expressed between stages.

**Additional file 11:** Comparison with nascent RNA.

**Additional file 12: Table S4.** Comparison between lifecycle stages of ChIP enrichment upstream of highly expressed genes differentially expressed between stages.

**Additional file 13: Fig S8.** Comparison of enrichment profiles of histones and their modifications across genes dynamically expressed in rings and trophozoites. Average log2 ratio of ChIP enrichment of Pf H2A.Z (green), H3K18ac (orange), H3K27ac (purple) and H3K4me1 (pink) all relative to ChIP enrichment of H3 plotted over the coding sequences ± 2500 bp of: gene set 3 (*n* = 299) that were expressed at least threefold more in trophozoite stage than in ring stage and that were in the top quartile by trophozoite-stage expression and: gene set 4 (*n* = 455) that were expressed at least threefold more in ring stage than in trophozoite stage and that were in the top quartile by ring-stage expression. 5′End: the start codon of a gene; 3′End: the stop codon of a gene.

**Additional file 14: Fig S9.** Comparison of enrichment profiles of histones and their modifications across genes dynamically expressed in trophozoites and schizonts. Average log2 ratio of ChIP enrichment of Pf H2A.Z (green), H3K18ac (orange), H3K27ac (purple) and H3K4me1 (pink) all relative to ChIP enrichment of H3 plotted over the coding sequences ± 2500 bp of: gene set 5 (*n* = 433) that were expressed at least threefold more in schizont stage than in trophozoite stage and that were in the top quartile by schizont-stage expression and: gene set 6 (*n* = 173) that were expressed at least threefold more in trophozoite stage than in schizont stage and that were in the top quartile by trophozoite-stage expression. 5′End: the start codon of a gene; 3′End: the stop codon of a gene.

**Additional file 15: Fig S10.** Enrichment of histones and their modifications across ATACseq peak summits. Log2 ChIP H3K4me1 (this study)/H3K4me3 [38], Log2 ChIP/input of H3K4me1, H3, H3K18ac, H3K27ac and Pf H2A.Z and ATACseq coverage normalised to gDNA [41] plotted across all schizont stage ATACseq peak summits ± 1800 bp. Average profile plots are shown on top, heatmaps below are all ranked in descending order of ATACseq coverage.

**Additional file 16: Fig S11.** Flow chart for the bioinformatic strategy used to identify candidate proximal and distal regulatory sequences upstream of schizont stage-expressed genes.

**Additional file 17: Table S5.** Coordinates of tandem ICPs tested for regulatory activity.

## Data Availability

The datasets generated and/or analysed during the current study are available in the NCBI Sequence Read Archive BioProject PRJNA612099, study SRP252482, BioSamples SAMN14361305 to SAMN14361313, accessions SRR11292854 to SRR11292897, Gene Expression Omnibus accession number GSE64691 [9], Gene Expression Omnibus accession number GSE104075 [41], NCBI Sequence Read Archive, accession number GSE80293 [8], Gene Expression Omnibus accession number GSE66185 [43], Gene Expression Omnibus accession number GSE68982 [42], Gene Expression Omnibus accession number GSE109599 [40].
