# Supplementary figures and images for: Histone modifications associated with gene expression and genome accessibility are dynamically enriched at Plasmodium falciparum regulatory sequences
Source: Epigenetics Chromatin. 2020 Nov 23;13:50. doi: 10.1186/s13072-020-00365-5 (PMC7682024; doi:10.1186/s13072-020-00365-5)

Additional figure S1

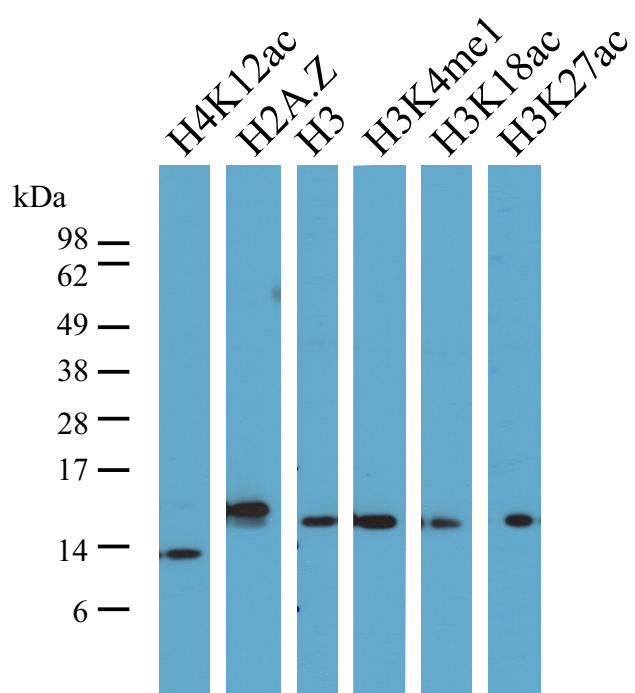

Supplement: Supplementary file 1 — Additional file 1: Fig S1. Specificity of ChIPseq antibodies for P. falciparum histones. Western blot of P. falciparum whole-cell lysate probed with the antibodies used in this study and with antibody to H4K12ac as an additional control showing the presence of multiple histones on the western blot. [file 13072_2020_365_MOESM1_ESM.pdf]

Additional figure S2

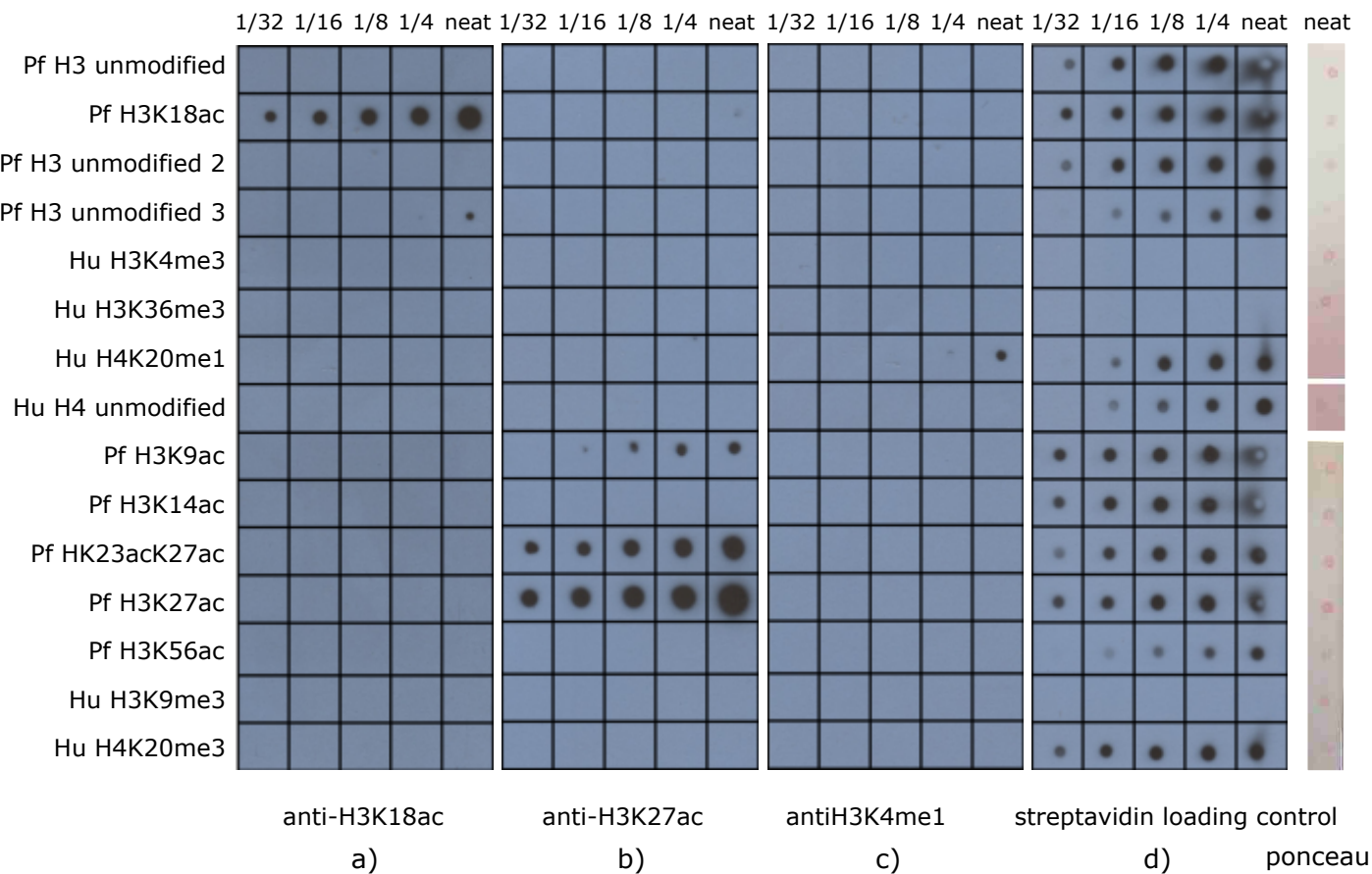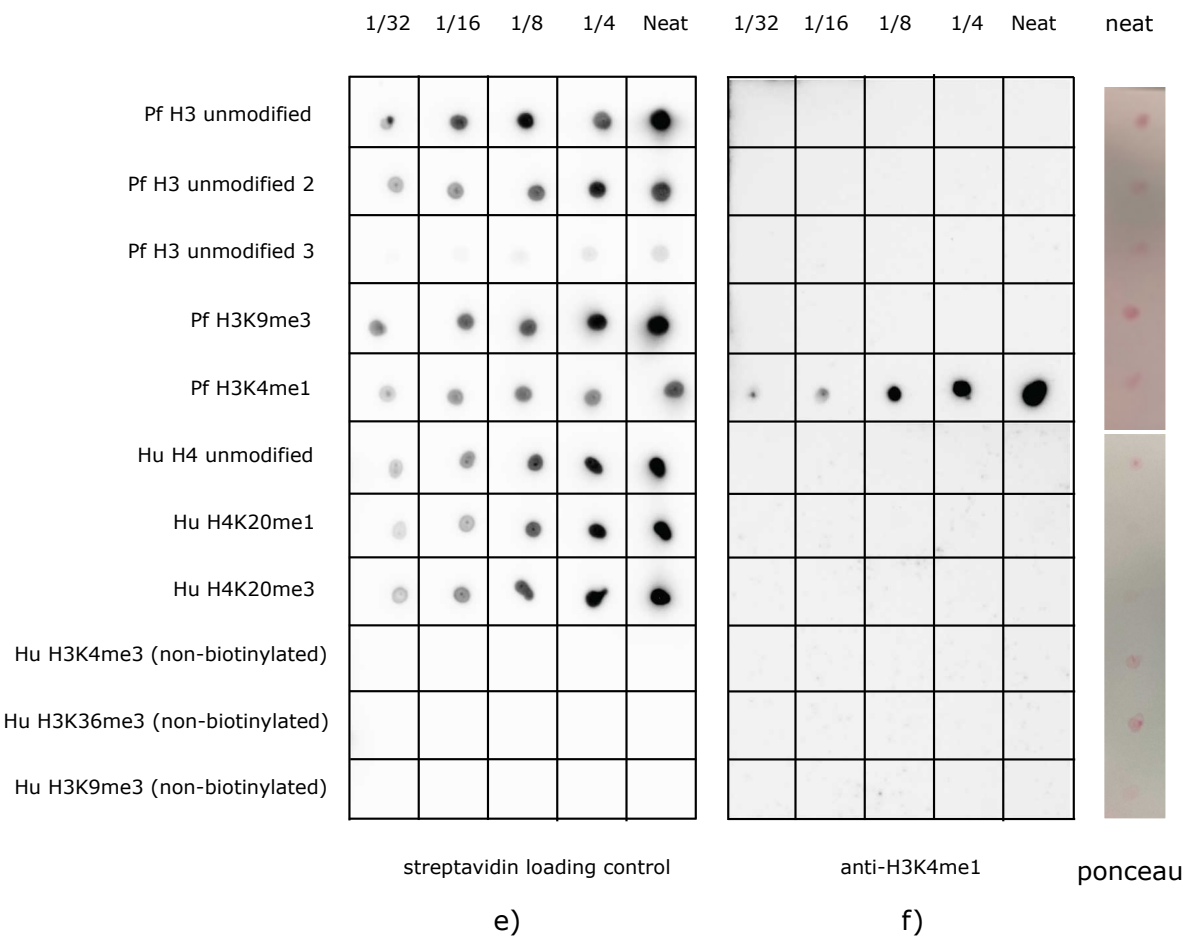

Supplement: Supplementary file 2 — Additional file 2: Fig S2. Specificity of ChIPseq antibodies for P. falciparum histone modifications. Dot blot of biotinylated histone peptides probed with rabbit antibody to a) H3K18ac (Abcam, Ab1191), b) rabbit antibody to H3K27ac (Abcam, Ab4729), c and f) rabbit antibody to H3K4me1(Abcam, Ab8895) and d and e) streptavidin as a loading control. Peptides indicated on the left were diluted from right to left as shown above the figure. Starting concentrations (neat) were 0.25 mg/ml for all P. falciparum (Pf) peptides and Human (Hu) H3K4me3, H3K36me3 and H3K9me3 peptides; 0.07 mg/ml for Human H4K20me1 and H4K20me3; 0.065 mg/ml for Human H4. Human H3K4me3, H3K36me3 and H3K9me3 were not biotinylated and their loading was confirmed by ponceau red staining shown for neat peptides in lanes to the left. P. falciparum H3 unmodified 1, 2 and 3 peptides represented residues 1–21, 21–43 and 47–65, respectively. A minor cross reaction of anti-H3K18ac antibody with a P. falciparum histone H3 unmodified peptide (residues 47–65) was observed, but the antibody was at least 32-fold more sensitive for H3K18ac than H3 (residues 47–65). The rabbit anti-H3K27ac antibody strongly bound both the P. falciparum H3K27ac peptide and the H3K23acK27ac peptide. The anti-H3K27ac antibody also bound the P. falciparum H3K9ac peptide but by densitometry it generated no more than 1/32 of the signal of H3K9ac. [file 13072_2020_365_MOESM2_ESM.pdf]

Additional figure S3

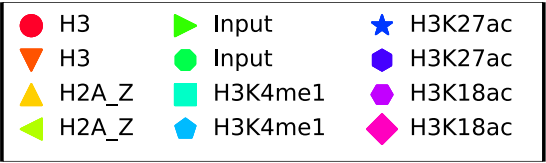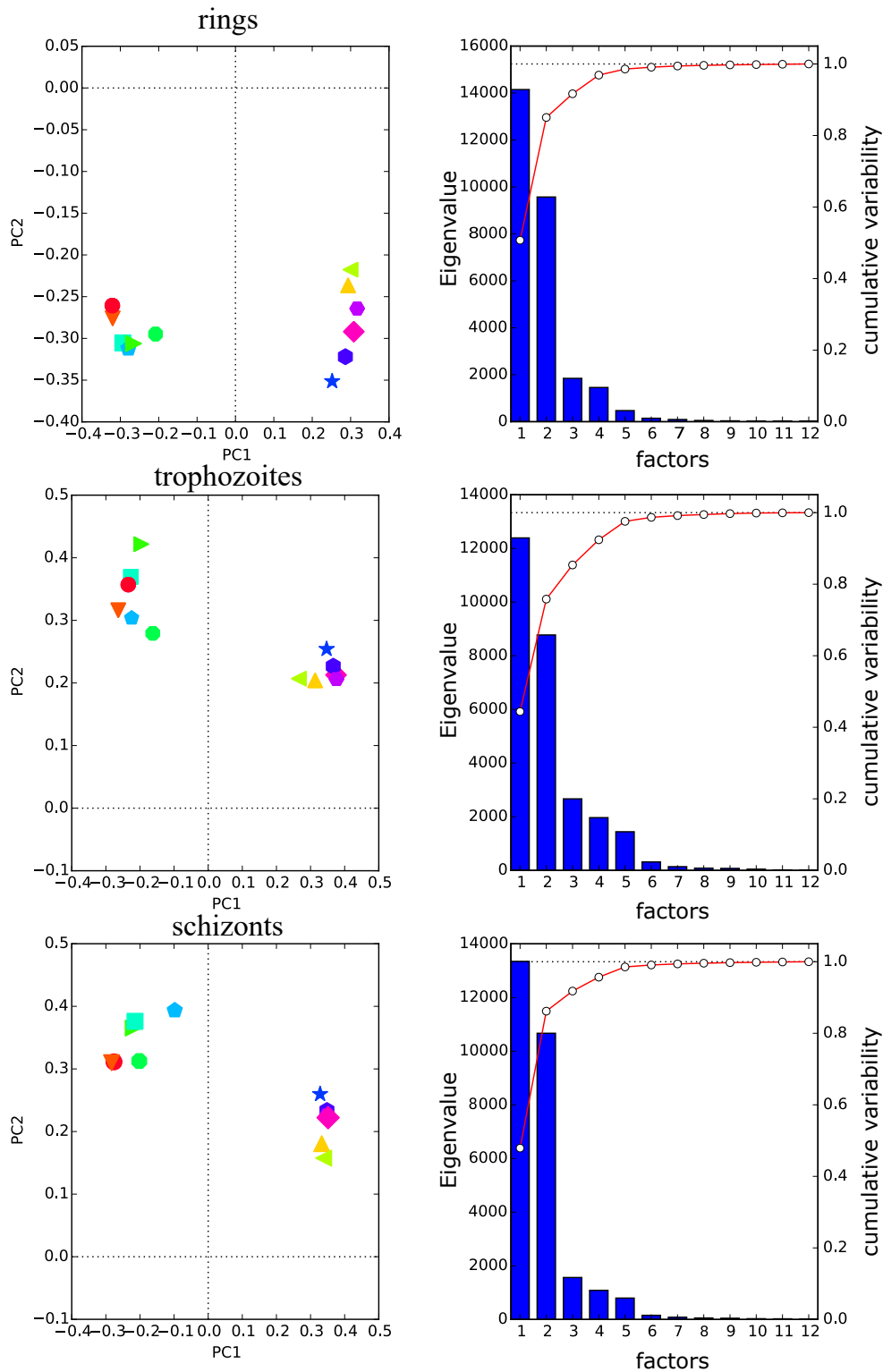

Supplement: Supplementary file 5 — Additional file 5: Fig S3. Reproducibility of ChIPseq. PCA plots and associated scree plots for all ChIPseq and input replicates for ring stages, trophozoite stages and schizont stages. [file 13072_2020_365_MOESM5_ESM.pdf]

Additional figure S4

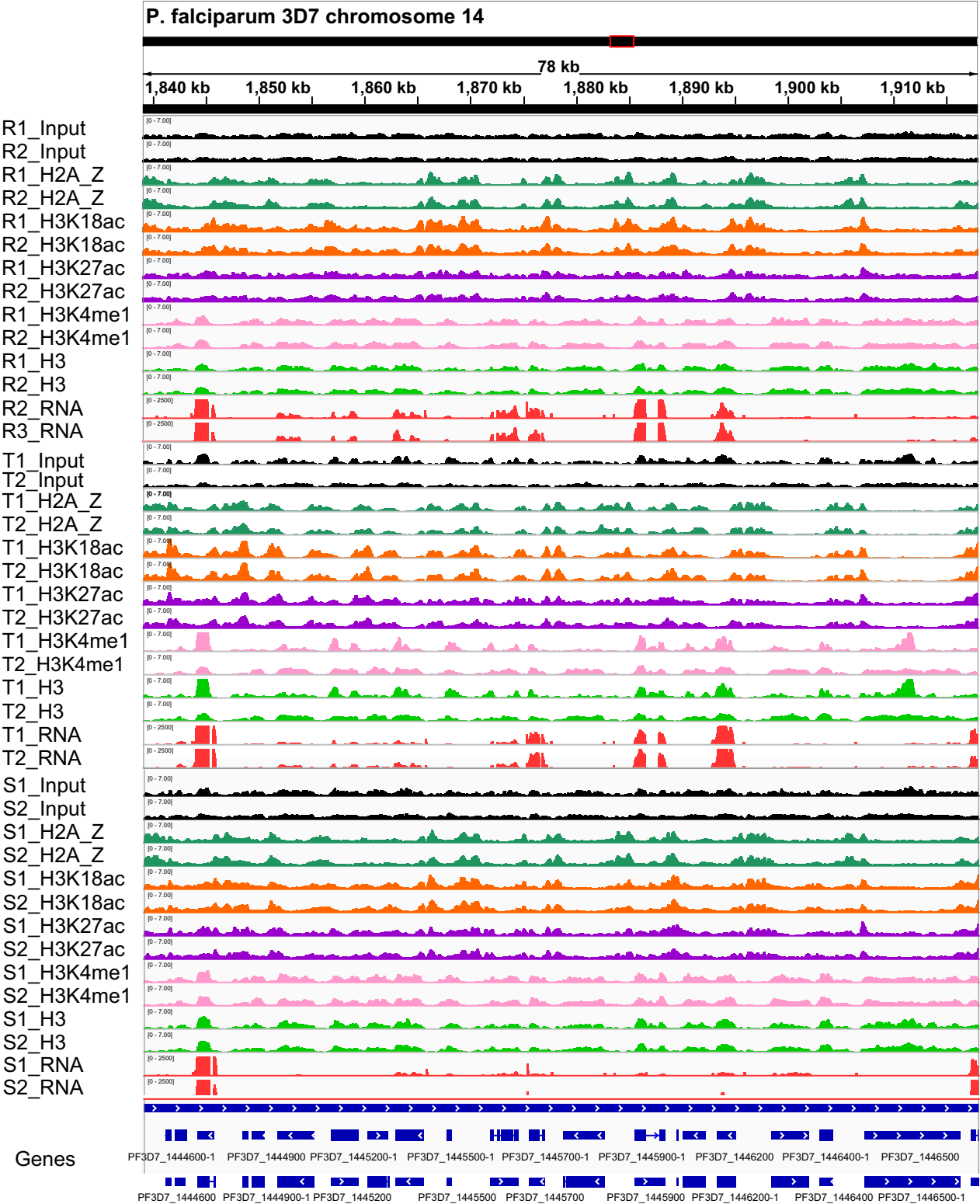

Supplement: Supplementary file 6 — Additional file 6: Fig S4. Individual sequencing tracks. Read coverage for ChIP, input and RNA sequencing replicates across 78 kb within chromosome 14. [file 13072_2020_365_MOESM6_ESM.pdf]

Additional figure S5

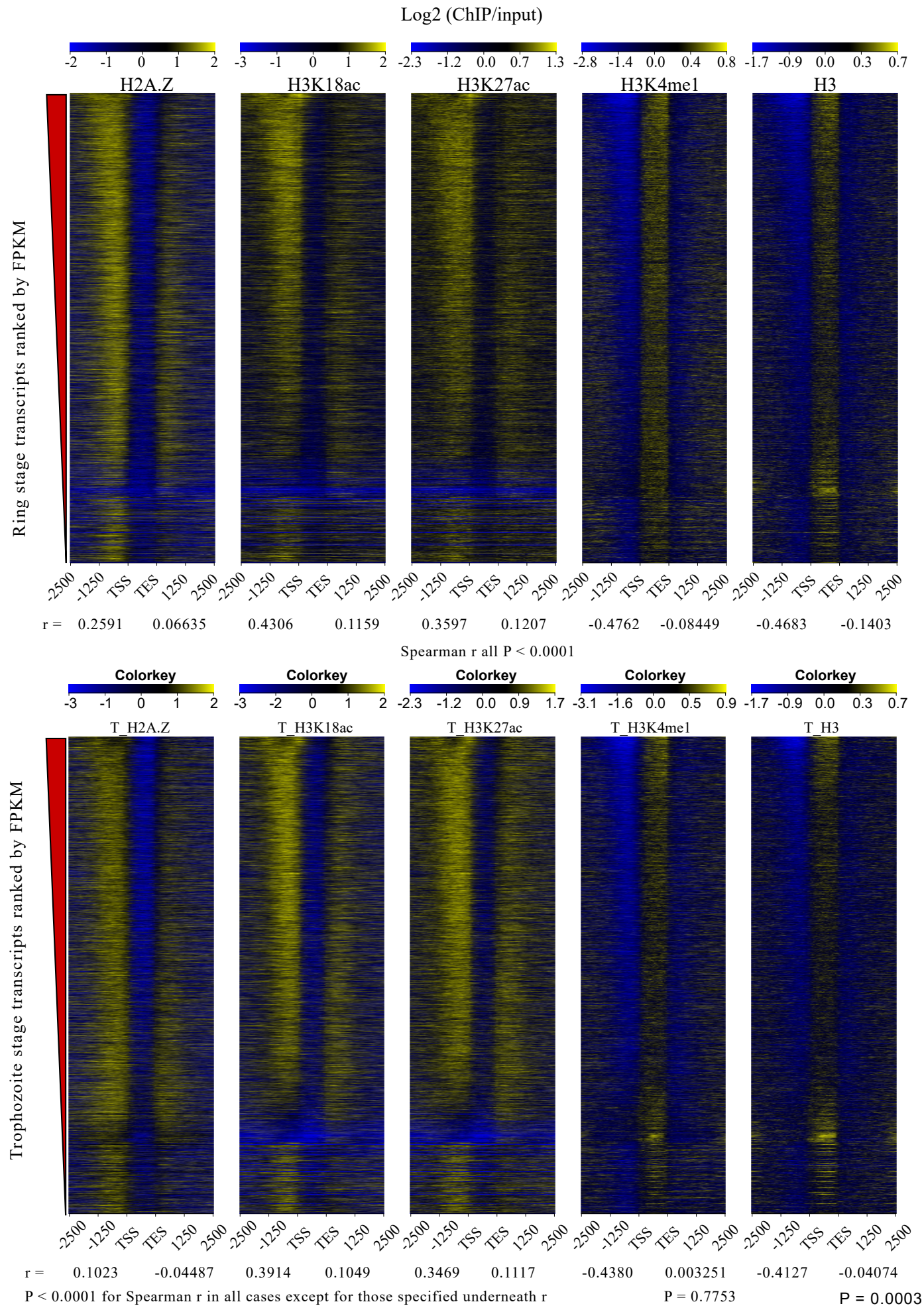

Supplement: Supplementary file 7 — Additional file 7: Fig S5. Correlations between expression level and histones and their modifications across transcriptional units. Log2 (ChIP/input) plotted from 2500 bp upstream (−2500) to 2500 bp downstream (2500) of the transcriptional start (TSS) and stop (TES) sites for 7612 ring stage transcripts and 7711 trophozoite stage transcripts assembled by Cufflinks ranked in descending order by transcript abundance (fpkm). The Spearman r correlation value (r) for the upstream and downstream enrichment of each histone or histone modification is indicated below the plots. [file 13072_2020_365_MOESM7_ESM.pdf]

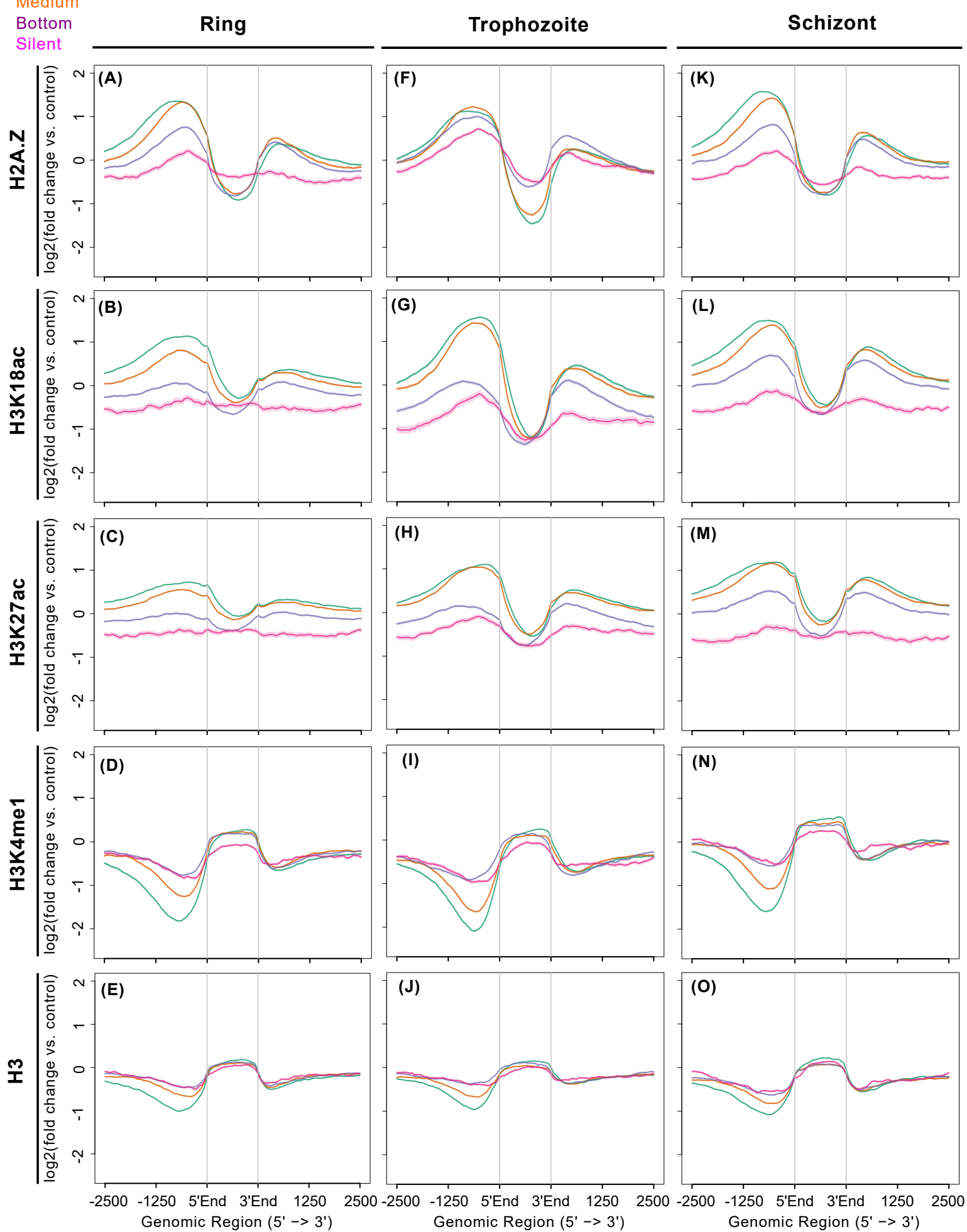

Supplement: Supplementary file 8 — Additional file 8: Fig S6. Average enrichment profiles of histones and their modifications across genes binned by expression level. The average profile of H2A.Z, H3K18ac, H3K27ac, H3K4me1 and H3 over the coding sequences ± 2500 bp for genes grouped into terciles by expression level (top green, middle orange, bottom purple) and for silent genes (pink). The enrichment profile of each chromatin mark was presented as the log2 ratio over the input control. In rings, trophozoites and schizonts gene expression categories were, respectively, the top (n = 1636, 1603, 1613), middle (n = 1495, 1539, 1519) and bottom (n = 1580, 1356, 1386) terciles, and silent genes (nn= 476, 530, 602). 5′End: the start codon of a gene; 3′End: the stop codon of a gene. [file 13072_2020_365_MOESM8_ESM.pdf]

Additional figure S7

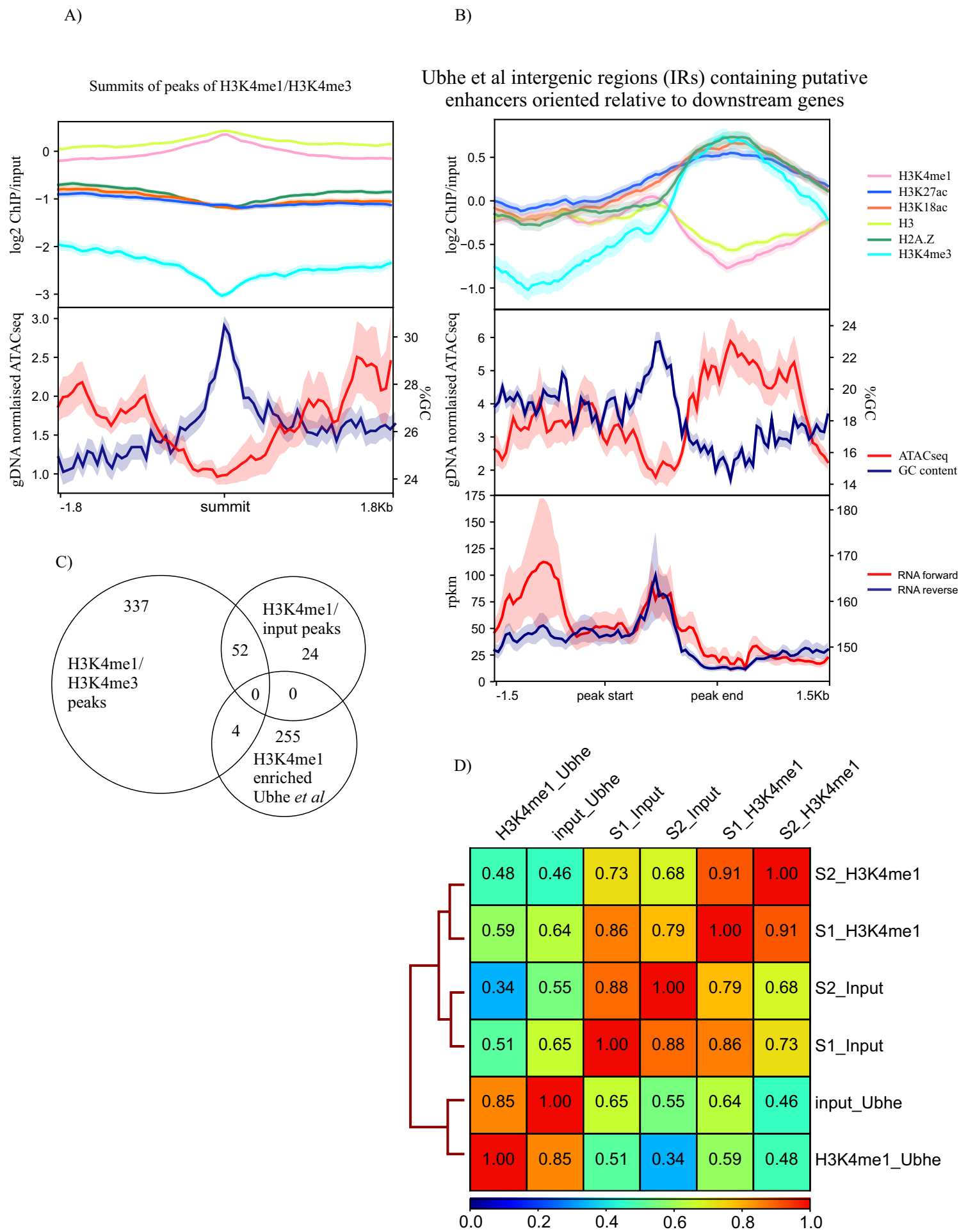

Supplement: Supplementary file 9 — Additional file 9: Fig S7. A) Average enrichment profiles of chromatin features and RNA levels from this study across the summits ± 1.8 kb of intergenic, peaks of H3K4me1 ChIP from this study relative to H3K4me3 from [38] B) Average enrichment profiles of chromatin features and RNA levels from this study across the intergenic peaks of H3K4me1 identified by Ubhe et al [48]. Average profile plots ± SE covering 1.5 kb up and downstream of published, “strong” H3K4me1 intergenic peaks longer than 1 bp (n = 259) [48] oriented according to the closest downstream gene. Top panel is the log2 ratio of ChIP over input average enrichment profiles of H3K4me1, H3K27ac, H3K18ac, H3 and Pf H2A.Z, middle panel is GC content and ATACseq normalised to gDNA and the bottom panel is RNA separated by strand. C) The number of intergenic peaks and their intersections from the H3K4me1/input from this study, the H3K4me1 from this study over the H3K4me3 from [38], and the H3K4me1 enriched intergenic regions from [48]. D) Spearman correlations for the single schizont H3K4me1 ChIP sample and matched input (Ubhe) analysed in [48] and for our two schizont replicates (S1 and S2) of H3K4me1 ChIP and matched input. [file 13072_2020_365_MOESM9_ESM.pdf]

Additional figure S8

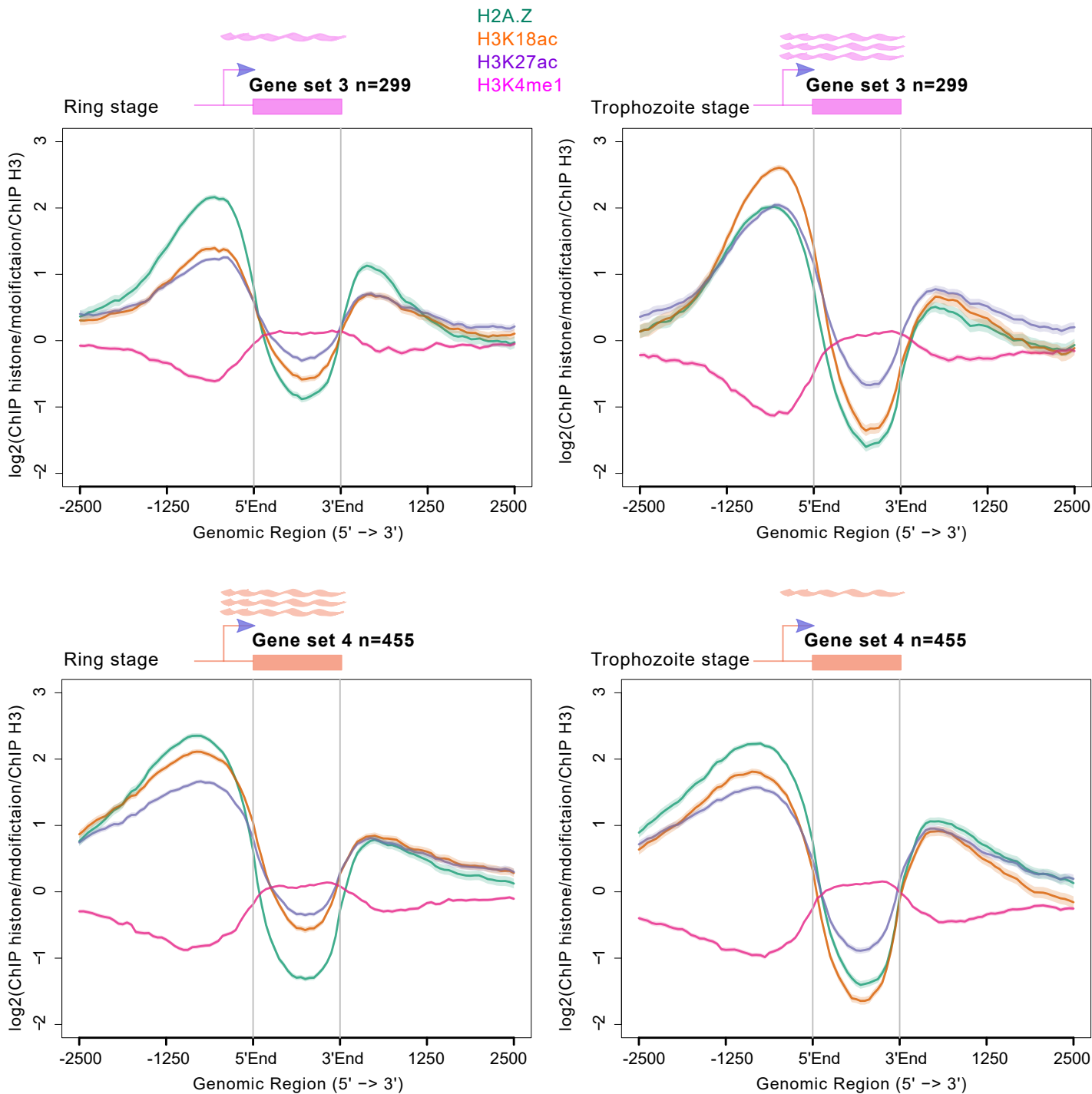

Supplement: Supplementary file 13 — Additional file 13: Fig S8. Comparison of enrichment profiles of histones and their modifications across genes dynamically expressed in rings and trophozoites. Average log2 ratio of ChIP enrichment of Pf H2A.Z (green), H3K18ac (orange), H3K27ac (purple) and H3K4me1 (pink) all relative to ChIP enrichment of H3 plotted over the coding sequences ± 2500 bp of: gene set 3 (n = 299) that were expressed at least threefold more in trophozoite stage than in ring stage and that were in the top quartile by trophozoite-stage expression and: gene set 4 (n = 455) that were expressed at least threefold more in ring stage than in trophozoite stage and that were in the top quartile by ring-stage expression. 5′End: the start codon of a gene; 3′End: the stop codon of a gene. [file 13072_2020_365_MOESM13_ESM.pdf]

Additional figure S9

H2A.Z  
H3K18ac  
H3K27ac  
H3K4me1

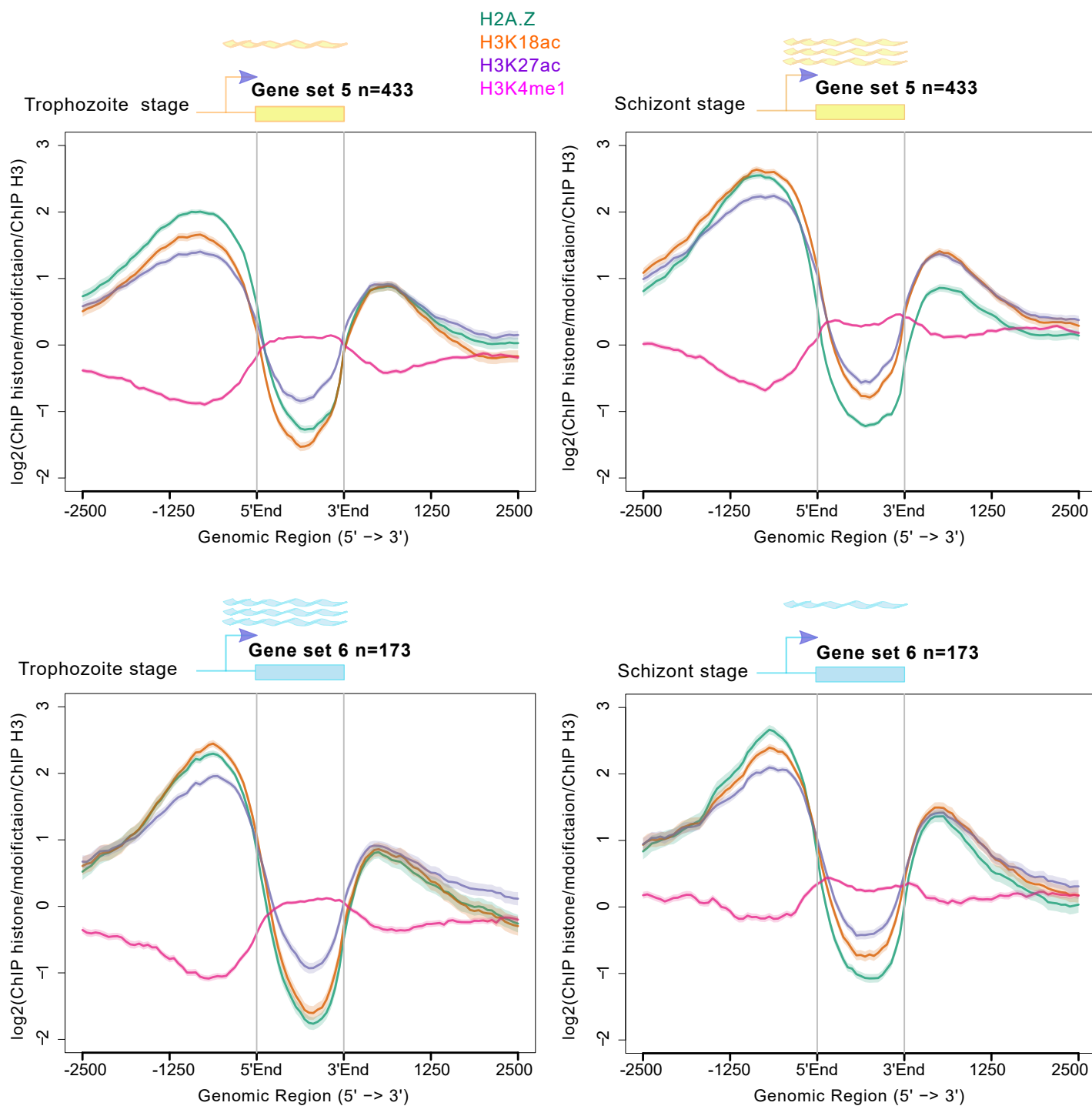

Supplement: Supplementary file 14 — Additional file 14: Fig S9. Comparison of enrichment profiles of histones and their modifications across genes dynamically expressed in trophozoites and schizonts. Average log2 ratio of ChIP enrichment of Pf H2A.Z (green), H3K18ac (orange), H3K27ac (purple) and H3K4me1 (pink) all relative to ChIP enrichment of H3 plotted over the coding sequences ± 2500 bp of: gene set 5 (n = 433) that were expressed at least threefold more in schizont stage than in trophozoite stage and that were in the top quartile by schizont-stage expression and: gene set 6 (n = 173) that were expressed at least threefold more in trophozoite stage than in schizont stage and that were in the top quartile by trophozoite-stage expression. 5′End: the start codon of a gene; 3′End: the stop codon of a gene. [file 13072_2020_365_MOESM14_ESM.pdf]

Additional figure S10

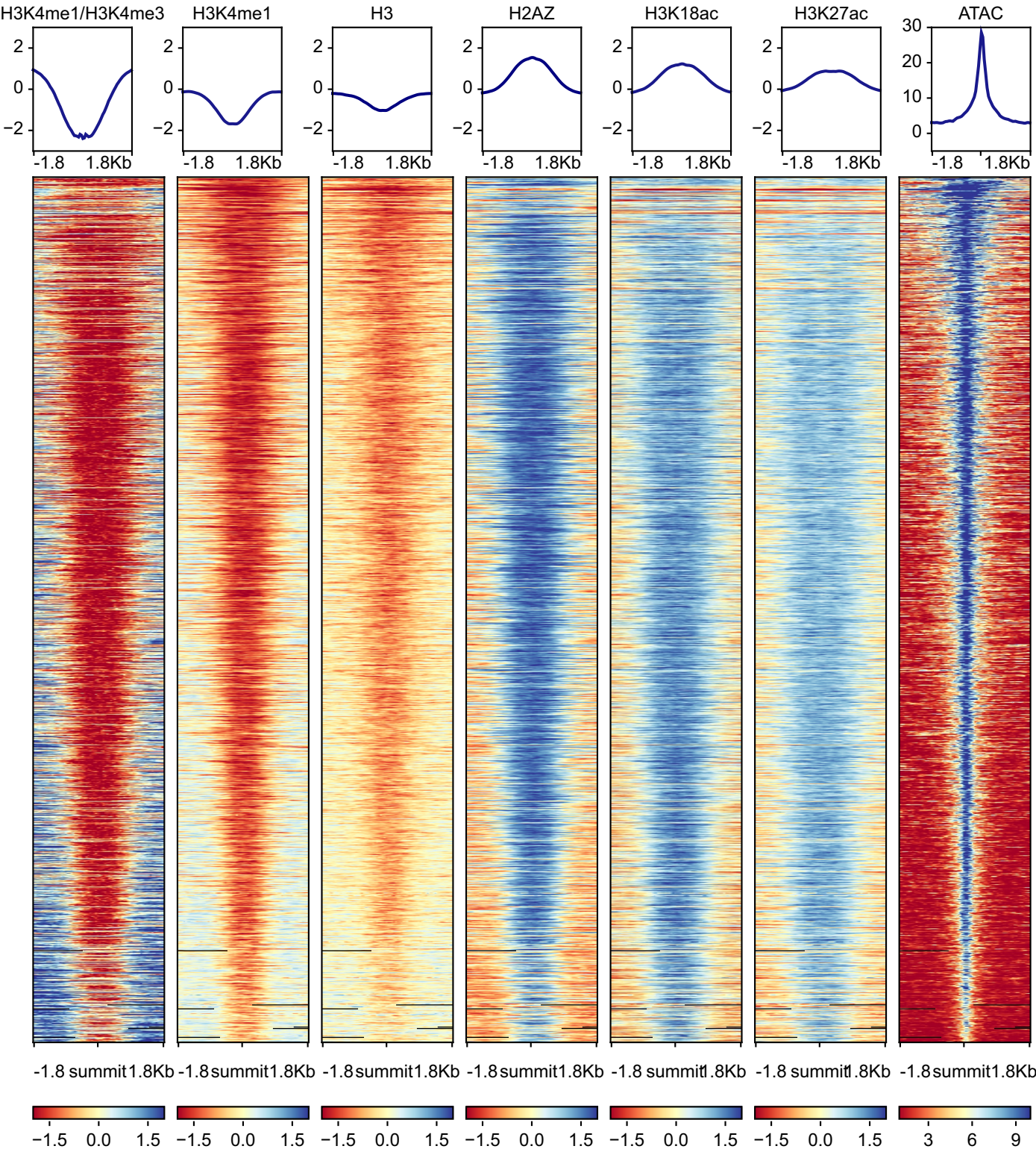

Schizont stage ATACseq peak summits

Supplement: Supplementary file 15 — Additional file 15: Fig S10. Enrichment of histones and their modifications across ATACseq peak summits. Log2 ChIP H3K4me1 (this study)/H3K4me3 [38], Log2 ChIP/input of H3K4me1, H3, H3K18ac, H3K27ac and Pf H2A.Z and ATACseq coverage normalised to gDNA [41] plotted across all schizont stage ATACseq peak summits ± 1800 bp. Average profile plots are shown on top, heatmaps below are all ranked in descending order of ATACseq coverage. [file 13072_2020_365_MOESM15_ESM.pdf]

Additional figure S11

call ChIP peaks  
(MACS2)

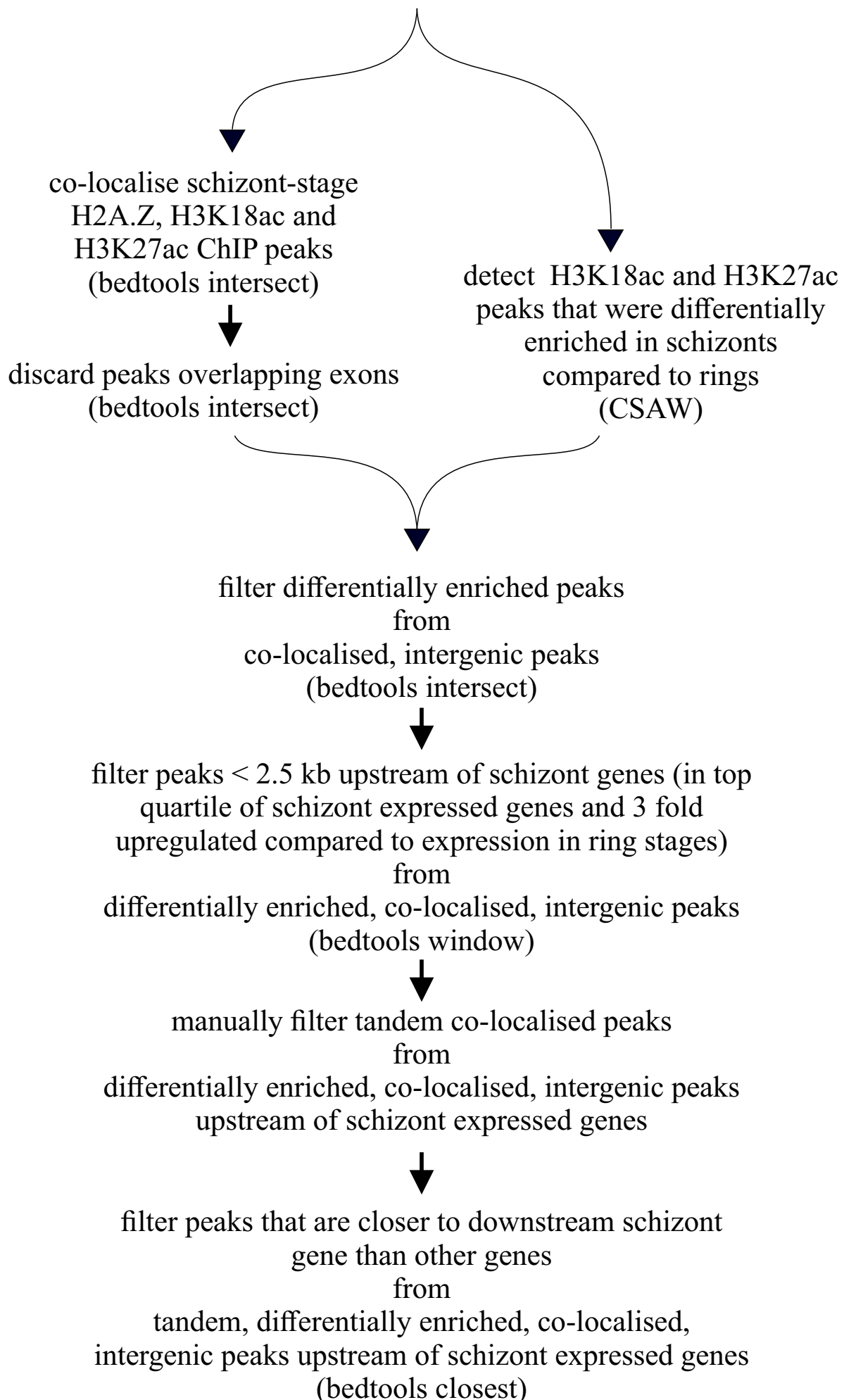

Supplement: Supplementary file 16 — Additional file 16: Fig S11. Flow chart for the bioinformatic strategy used to identify candidate proximal and distal regulatory sequences upstream of schizont stage-expressed genes. [file 13072_2020_365_MOESM16_ESM.pdf]
