# Supplementary material for: Histone modifications associated with gene expression and genome accessibility are dynamically enriched at Plasmodium falciparum regulatory sequences
Source: Epigenetics Chromatin. 2020 Nov 23;13:50. doi: 10.1186/s13072-020-00365-5 (PMC7682024; doi:10.1186/s13072-020-00365-5)
Supplement: Supplementary file 3 — Additional file 3: Table S1. Quality summary of ChIPseq data. [file 13072_2020_365_MOESM3_ESM.docx]

| Sample | %≥Q30 | Read pairs  Processed | Read pairs mapped | % read pairs mapped | Read pairs correctly paired | % read pairs correctly paired | Indels | Sequencing depth |
| --- | --- | --- | --- | --- | --- | --- | --- | --- |
| R1_Input | 87.10 | 2865281 | 2848095 | 99.40 | 2609070 | 91.61 | 120729 | 31.4 |
| R1_H2A.Z | 80.00 | 2695666 | 2671973 | 99.10 | 2382045 | 89.15 | 152601 | 29.5 |
| R1_H3K18ac | 81.25 | 2426149 | 2404810 | 99.10 | 2097744 | 87.23 | 136930 | 26.6 |
| R1_H3K27ac | 84.84 | 2558154 | 2544905 | 99.50 | 2293007 | 90.10 | 130836 | 28.0 |
| R1_H3K4me1 | 88.90 | 2682661 | 2669065 | 99.50 | 2452758 | 91.90 | 98055 | 29.4 |
| R1_H3 | 88.36 | 2803932 | 2786329 | 99.40 | 2558449 | 91.82 | 106845 | 30.7 |
| T1_Input | 86.37 | 4860481 | 4804838 | 98.90 | 4442642 | 92.46 | 184132 | 53.3 |
| T1_H2A.Z | 85.00 | 5338249 | 5304519 | 99.40 | 4888577 | 92.16 | 243300 | 58.5 |
| T1_H3K18ac | 84.13 | 4992193 | 4958742 | 99.30 | 4508624 | 90.92 | 223397 | 54.7 |
| T1_H3K27ac | 84.86 | 4557747 | 4510498 | 99.00 | 4126932 | 91.50 | 206879 | 49.9 |
| T1_H3K4me1 | 85.73 | 4464765 | 4424764 | 99.10 | 4070817 | 92.00 | 139058 | 48.9 |
| T1_H3 | 85.14 | 4431018 | 4384103 | 98.90 | 4002021 | 91.28 | 156577 | 48.5 |
| S1_Input | 88.02 | 2476442 | 2457917 | 99.30 | 2242950 | 91.25 | 106481 | 27.1 |
| S1_H2A.Z | 82.25 | 2741401 | 2720635 | 99.20 | 2435779 | 89.53 | 156639 | 30.0 |
| S1_H3K18ac | 81.45 | 2553281 | 2536029 | 99.30 | 2240387 | 88.34 | 148588 | 28.0 |
| S1_H3K27ac | 79.33 | 3559901 | 3495439 | 98.20 | 3099809 | 88.68 | 173985 | 39.0 |
| S1_H3K4me1 | 90.60 | 2773002 | 2758415 | 99.50 | 2574121 | 93.32 | 96126 | 30.4 |
| S1_H3 | 89.00 | 2714039 | 2699601 | 99.50 | 2456440 | 90.99 | 96514 | 29.7 |
| R2_Input | 90.45 | 5248468 | 5221178 | 99.50 | 4895777 | 93.77 | 211108 | 57.5 |
| R2_H2A.Z | 82.82 | 4366495 | 4318566 | 98.90 | 3870498 | 89.62 | 225346 | 47.8 |
| R2_H3K18ac | 85.40 | 3766574 | 3721459 | 98.80 | 3320430 | 89.22 | 191327 | 41.3 |
| R2_H3K27ac | 87.50 | 4757483 | 4726410 | 99.30 | 4347576 | 91.98 | 214893 | 52.1 |
| R2_H3K4me1 | 92.12 | 5248647 | 5222090 | 99.50 | 4893238 | 93.70 | 166251 | 57.5 |
| R2_H3 | 90.99 | 4759686 | 4732623 | 99.40 | 4425905 | 93.52 | 169165 | 52.1 |
| T2_Input | 89.89 | 4871448 | 4841089 | 99.40 | 4297788 | 88.78 | 203182 | 53.4 |
| T2_H2A.Z | 83.86 | 4044910 | 4006127 | 99 | 3368264 | 84.08 | 214628 | 44.3 |
| T2_H3K18ac | 82.74 | 3782333 | 3750494 | 99.20 | 3033026 | 80.87 | 205148 | 41.4 |
| T2_H3K27ac | 85.18 | 3537474 | 3495988 | 98.80 | 2940206 | 84.10 | 197122 | 38.8 |
| T2_H3K4me1 | 91.57 | 3796880 | 3771055 | 99.30 | 3362766 | 89.17 | 120798 | 41.6 |
| T2_H3 | 90.62 | 4375310 | 4353187 | 99.50 | 3846122 | 88.35 | 165446 | 47.9 |
| S2_Input | 90.69 | 5275783 | 5242656 | 99.40 | 4885985 | 93.20 | 200813 | 57.8 |
| S2_H2A.Z | 82.11 | 4002458 | 3966456 | 99.10 | 3423838 | 86.32 | 212855 | 43.9 |
| S2_H3K18ac | 85.40 | 4100724 | 4067638 | 99.20 | 3508249 | 86.25 | 221163 | 44.9 |
| S2_H3K27ac | 84.99 | 4664307 | 4628129 | 99.20 | 4038037 | 87.25 | 232903 | 51.1 |
| S2_H3K4me1 | 91.15 | 4769961 | 4743103 | 99.40 | 4284729 | 90.34 | 168074 | 52.3 |
| S2_H3 | 90.34 | 4514148 | 4488426 | 99.40 | 4144211 | 92.33 | 156964 | 49.5 |

Table S1 Quality summary of ChIPseq data
