# Supplementary material for: Histone modifications associated with gene expression and genome accessibility are dynamically enriched at Plasmodium falciparum regulatory sequences
Source: Epigenetics Chromatin. 2020 Nov 23;13:50. doi: 10.1186/s13072-020-00365-5 (PMC7682024; doi:10.1186/s13072-020-00365-5)
Supplement: Supplementary file 4 — Additional file 4: Table S2. Quality summary of RNAseq data. [file 13072_2020_365_MOESM4_ESM.docx]

| Sample | %≥Q30 | Input read pairs | left reads mapped | right reads mapped | % reads aligned | Read pairs aligned | % read pairs correctly paired | % read pairs with concordant alignment | Sequencing depth |
| --- | --- | --- | --- | --- | --- | --- | --- | --- | --- |
| R1_RNAseq | 91.29 | 2154273 | 2005089 | 2005437 | 93.10 | 1937972 | 89.96 | 89.30 | 23.6 |
| R2_RNAseq | 96.63 | 3008305 | 2814508 | 2828472 | 93.80 | 2732186 | 90.82 | 90.70 | 33.0 |
| T1_RNAseq | 94.09 | 2595019 | 2444098 | 2440529 | 94.10 | 2376987 | 91.60 | 91.20 | 28.4 |
| T2_RNAseq | 95.98 | 1333638 | 1262151 | 1264840 | 94.70 | 1229492 | 92.19 | 91.90 | 14.6 |
| T3_RNAseq | 97.80 | 3307338 | 3140869 | 3149824 | 95.10 | 3062304 | 92.59 | 92.50 | 36.2 |
| S1_RNAseq | 93.20 | 1391205 | 1307289 | 1304643 | 93.90 | 1269710 | 91.27 | 90.80 | 15.2 |
| S2_RNAseq | 95.48 | 2339059 | 2211554 | 2216668 | 94.70 | 2156474 | 92.19 | 91.90 | 25.6 |
| S3_RNAseq | 97.86 | 3235045 | 3070531 | 3076170 | 95.00 | 2992622 | 92.51 | 92.40 | 35.4 |

Table S2 Quality summary of RNAseq data
