# Supplementary material for: Histone modifications associated with gene expression and genome accessibility are dynamically enriched at Plasmodium falciparum regulatory sequences
Source: Epigenetics Chromatin. 2020 Nov 23;13:50. doi: 10.1186/s13072-020-00365-5 (PMC7682024; doi:10.1186/s13072-020-00365-5)
Supplement: Supplementary file 10 — Additional file 10: Table S3. Number of highly expressed genes differentially expressed between stages. [file 13072_2020_365_MOESM10_ESM.docx]

| Genes expression profile | Number genes |
| --- | --- |
| Top expression quartile in Schizonts 3X ≥ rings | 478 |
| Top expression quartile in Rings 3X ≥ schizonts | 528 |
| Top expression quartile in Trophozoites 3X ≥ rings | 299 |
| Top expression quartile in Rings 3X ≥ trophozoites | 455 |
| Top expression quartile in Schizonts 3X ≥ trophozoites | 433 |
| Top expression quartile in Trophozoites 3X ≥ schizonts | 173 |

Table S3 Number of highly expressed genes differentially expressed between stages
