# Supplementary material for: Histone modifications associated with gene expression and genome accessibility are dynamically enriched at Plasmodium falciparum regulatory sequences
Source: Epigenetics Chromatin. 2020 Nov 23;13:50. doi: 10.1186/s13072-020-00365-5 (PMC7682024; doi:10.1186/s13072-020-00365-5)
Supplement: Supplementary file 11 — Additional file 11: Comparison with nascent RNA. [file 13072_2020_365_MOESM11_ESM.pdf]

Additional file 11, comparison with nascent RNA

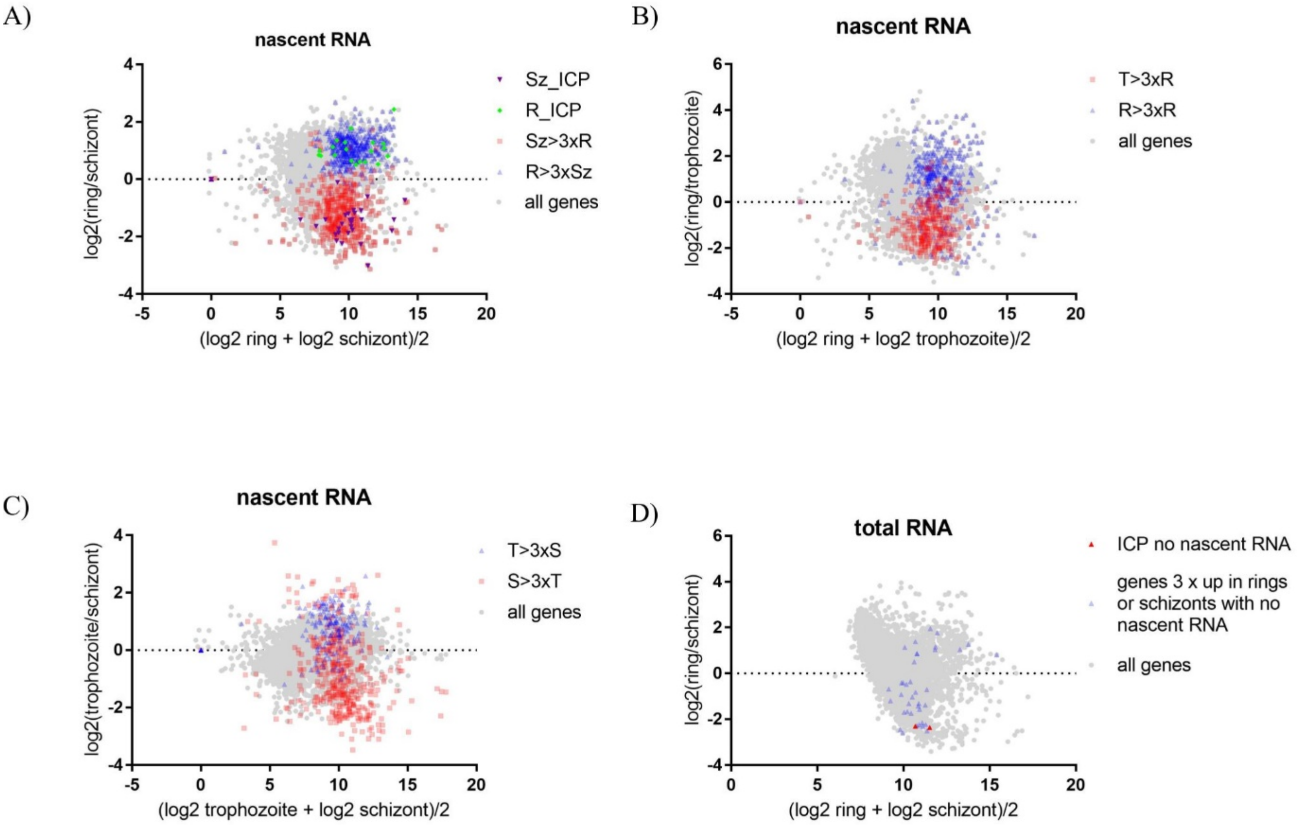

legend: MA plots; pairwise comparisons of nascent RNA or matched total RNA levels from [1] between: A & D) 11 hours post invasion rings stages and 41 hpi schizonts, B) 11 hpi ring stages and 33 hpi trophozoites, C) 33 hpi trophozoites and 41 hpi schizonts. A) Genes by the current RNAseq analysis that were in the top quartile by expression in rings and 3 fold more highly expressed in rings compared to schizonts (R>3xSz), in the top quartile by expression in schizonts and 3 fold more highly expressed in schizonts than rings (Sz>3xR), that were downstream of tandem, intergenic, co-localised peaks (ICPs) of H3K18ac, H3K27ac and P. falciparum H2A.Z in schizonts (Sz_ICP) or rings (R_ICP), B) Genes by the current RNAseq analysis that were in the top quartile by expression in rings and 3 fold more highly expressed in rings compared to trophozoites (R>3xT), in the top quartile by expression in trophozoites and 3 fold more highly expressed in trophozoites than rings (T>3xR), C) Genes by the current RNAseq analysis that were in the top quartile by expression in trophozoites and 3 fold more highly expressed in trophozoites compared to schizonts (T>3xS), in the top quartile by expression in schizonts and 3 fold more highly expressed in schizonts than trophozoites (S>3xT), D) two genes downstream of ICPs in schizonts that had no nascent RNA throughout the lifecycle, (ICP no nascent RNA), 40 genes that were in the top quartile of expression in either rings or schizonts and 3 fold more expressed in rings or schizonts than in the other stage but that had no nascent RNA throughout the lifecycle (genes 3 x up in rings or schizonts with no nascent RNA).

To confirm the validity of the dynamically regulated gene-sets that we defined by RNAseq we analysed the “in vivo” nascent RNAseq dataset of Painter *et al* [1] for ring stages at 11 hours

post invasion (hpi), trophozoites (33 hpi) and schizonts (41 hpi), these being the midpoints of our 6 h windows for the same stages. Our dynamically regulated gene-sets (Fig 5, Fig S8, Fig S9) had higher median expression than the entire gene repertoire in the nascent RNA dataset by Mann Whitney test for each of our 6 compared gene-sets (median and p values for gene-set 1 schizonts 1251 vs 414 $p < 0.0001$, gene-set 2 rings median 651 vs 420 $p<0.0001$, gene-set 3 trophozoites 915 vs 459 $p<0.0001$, gene-set 4 rings 1277 vs 420 $p<0.0001$, gene-set 5 schizonts 1142 vs 414 $p<0.0001$, gene-set 6 trophozoites 738 vs 459 $p<0.0001$).

We plotted pairwise comparisons of the nascent RNA microarray data for ring stages at 11 hours post invasion (hpi), trophozoites (33 hpi) and schizonts (41 hpi) in three MA plots (Supplementary material 1 A, B, C). We indicated on these plots the genes we had identified as dynamically regulated by RNAseq. Nearly all of the genes we identified as dynamically regulated by RNAseq were also dynamically regulated in the same direction in the nascent RNA dataset in the ring vs schizont comparison (Supplementary material 1A) and the trophozoite vs rings comparison (Supplementary material 1B). The trophozoite and schizont gene-sets were also clearly separated in the predicted directions but there was more overlap between them than in the other two comparisons (Supplementary material 1C). This was consistent with our sampling windows, rings 8-14 hours post invasion, trophozoites 30-36 hpi and schizonts 38-44 hpi, thus the 2h window of separation was much shorter for the trophozoite to schizont comparison than for the other two comparisons (12 h and 16 h). We specifically looked for the 30 genes dynamically expressed in schizont stages and the 24 genes dynamically expressed in ring stages that had tandem intergenic colocalised peaks (ICPs) of H3K18ac, H3K27ac and Pf H2A.Z upstream and were used to define putative regulatory sequences. These genes were all abundantly expressed as nascent mRNAs in the stage predicted by our RNAseq with the exception of two schizont stage genes that had zero nascent transcripts throughout the entire lifecycle (Supplementary material 1A). These two genes were however abundantly transcribed in the total RNA matched dataset from the Painter *et al* nascent RNA study (Supplementary material 1D) and so must have been missed as nascent transcripts due to technical error.

To investigate the variation we looked in more detail at the rings versus schizonts comparison, as these were the stages we used to define putative promoters and enhancers by bioinformatic means. Forty of the 998 genes that we defined as dynamically regulated by RNAseq in the comparison of rings and schizonts had 0 nascent transcripts in all 48 of the 1 h windows of the lifecycle but were abundant transcripts in the total RNA dataset from matched stages from the same nascent RNA study (Supplementary material 1D), i.e. the nascent RNA study failed to detect the nascent transcripts of these genes. In fact the nascent RNA dataset contains 219 genes that have zero levels of nascent transcript at any of the 1 h windows sampled across the 48 h lifecycle yet steady state transcripts are detected for all genes and many of these genes lacking any nascent transcripts are very abundantly transcribed (Supplementary material 1D), e.g. cen H3.

1. Painter HJ, Chung NC, Sebastian A, Albert I, Storey JD, Llinas M. Genome-wide real-time in vivo transcriptional dynamics during Plasmodium falciparum blood-stage development. Nat Commun. 2018;9:2656. https://doi.org/10.1038/s41467-018-04966-3.
