# Supplementary material for: Histone modifications associated with gene expression and genome accessibility are dynamically enriched at Plasmodium falciparum regulatory sequences
Source: Epigenetics Chromatin. 2020 Nov 23;13:50. doi: 10.1186/s13072-020-00365-5 (PMC7682024; doi:10.1186/s13072-020-00365-5)
Supplement: Supplementary file 12 — Additional file 12: Table S4. Comparison between lifecycle stages of ChIP enrichment upstream of highly expressed genes differentially expressed between stages. [file 13072_2020_365_MOESM12_ESM.docx]

|  | **Gene set** | **H2A.Z** | **H3K18ac** | **H3K27ac** | **H3K4me1** |
| --- | --- | --- | --- | --- | --- |
| Ring vs. Schizonts | 1 (schizont) | P<0.0001 | P<0.0001 | P<0.0001 | P<0.0001 |
|  | 2 (ring) | P<0.0001 | P=0.0075 | P<0.0001 | P<0.0001 |
| Ring vs. Trophozoite | 3 (ring) | P=0.0002 | P<0.0001 | P<0.0001 | P<0.0001 |
|  | 4 (trophozoite) | P<0.0001 | P<0.0001 | P<0.0001 | P<0.0001 |
| Trophozoite vs. Schizonts | 5 (schizont) | P<0.0001 | P<0.0001 | P<0.0001 | P<0.0001 |
|  | 6 (trophozoite) | P<0.0001 | P=0.1325 | P<0.0001 | P<0.0001 |

Table S4. The mean enrichment of log2 ((ChIP H2AZ or H3 modification)/ChIP H3) in each of twenty-one continuous windows from the start codon to 1250 bp upstream for each gene in a gene set for a lifecycle stage were extracted and the mean across all windows calculated. The columns of mean values from the genes in a set were compared between stages by Wilcoxon matched-pairs signed rank test.
