# Supplementary material for: Histone modifications associated with gene expression and genome accessibility are dynamically enriched at Plasmodium falciparum regulatory sequences
Source: Epigenetics Chromatin. 2020 Nov 23;13:50. doi: 10.1186/s13072-020-00365-5 (PMC7682024; doi:10.1186/s13072-020-00365-5)
Supplement: Supplementary file 17 — Additional file 17: Table S5. Coordinates of tandem ICPs tested for regulatory activity. [file 13072_2020_365_MOESM17_ESM.docx]

| Gene |  | Distal upstream ICP | Proximal upstream ICP |
| --- | --- | --- | --- |
| PF3D7_1362000 | schizont | 2483625 > 2484591 | 2485288 > 2486753 |
| PF3D7_1460600 | schizont | 2471728 > 2472587 | 2473101 > 2473757 |
| PF3D7_0920700 | schizont | 3D7_09_v3 847929 > 848449 | 848865 > 849514 |
| PF3D7_1037300 | ring | 3D7_10_v3 1480916 > 1481454 | 1478814 > 1480522 |
| PF3D7_0703500 | ring | 3D7_07_v3 132287 > 134041 | 134515 > 135605 |
| PF3D7_1453900 | ring | 3D7_07_v3 2212914 > 2213547 | 2213942 > 2214746 |
| PF3D7_1370000 | Control* |  | 3D7_13_v3 2782111 > 2782904 |

Additional TableS5. Coordinates of tandem ICPs tested for regulatory activity. Coordinates were from *P. falciparum* 3D7 strain genome release 12 version 3 (PlasmoDB). Sequences were tested for regulatory activity in transient transfection of nanoluciferase reporter construct assays. Genes downstream and adjacent to the ICPs are indicated as are the lifecycle stage in which the genes were in the top quartile by expression and during which they were expressed at least three-fold higher than in the other stage compared. * The control sequence was an AT matched intergenic region that was located between two tail to tail oriented tRNA genes, Pf3D7_1370000 and PF3D7_1370100.
